# USP39 promotes malignant proliferation and angiogenesis of renal cell carcinoma by inhibiting VEGF-A_165b_ alternative splicing via regulating SRSF1 and SRPK1

**DOI:** 10.1186/s12935-021-02161-x

**Published:** 2021-09-20

**Authors:** Xiu-wu Pan, Da Xu, Wen-jin Chen, Jia-xin Chen, Wei-jie Chen, Jian-qing Ye, Si-shun Gan, Wang Zhou, Xu Song, Lei Shi, Xin-gang Cui

**Affiliations:** 1 Department of Urology, Xinhua Hospital, School of Medicine, Shanghai Jiaotong University, 1665 Kongjiang Road, Shanghai, 200092 China; 2 Depanrtment of Urology, Third Affiliated Hospital of the Second Military Medical University, Shanghai, 201805 China; 3 Department of Urology, Shanghai Seventh People’s Hospital, Shandong, 200137 China; 4 Department of Urology, Yantai Yuhuangding Hospital of Qingdao University Medical College, Shandong, 264000 China

**Keywords:** Renal cell carcinoma, USP39, VEGF-A alternative splicing, SRPK1, SRSF1

## Abstract

**Background:**

The benefit of targeted therapy for renal cell carcinoma (RCC) is largely crippled by drug resistance. Rapid disease progression and poor prognosis occur in patients with drug resistance. New treatments demand prompt exploration for clinical therapies. Ubiquitin-specific peptidase 39 (USP39) serves as the pro-tumor factor in several previous studies of other malignant tumors. To investigate the function and mechanism of USP39 in promoting malignant proliferation and angiogenesis of RCC.

**Methods:**

We applied ONCOMINE database to analyze the correlation between USP39 expression level and the clinical characteristics of RCC. USP39 knockdown or overexpression plasmids were transfected into 786-O and ACHN cells. The HUVEC received cell supernatants of 786-O and ACHN cells with knockdown or overexpression USP39.The effect of USP39 on RCC was evaluated by MTT assay, cell cycle analysis, colony formation assay and tubule formation assay. The interaction between USP39 and VEGF-A alternative splicing was assessed by affinity purification and mass spectrometry, co-immunoprecipitation and Western blot assays.

**Results:**

The mRNA expression level of USP39 in RCC was significantly higher than that in normal renal tissue (P < 0.001), and negatively correlated with the survival rate of RCC patients (P < 0.01). Silencing of USP39 in 786-O and ACHN cells inhibited cell proliferation and colony formation, and induced S phase arrest. USP39 overexpression significantly increased the number of tubules (P < 0.05) and branches (P < 0.01) formed by HUVEC cells, and USP39 knockdown produced an opposite effect (P < 0.05). The USP39 _(101–565)_ fragment directly mediated its binding to SRSF1 and SRPK1, and promoted the phosphorylation of SRSF1 to regulate VEGF-A alternative splicing. USP39 knockdown upregulated the expression of VEGF-A_165b_, and USP39 overexpression downregulated the expression of VEGF-A_165b_ significantly (both P < 0.05).

**Conclusion:**

USP39 acted as a pro-tumor factor by motivating the malignant biological processes of RCC, probably through inhibiting VEGF-A^165b^ alternative splicing and regulating SRSF1 and SRPK1. USP39 may prove to be a potential therapeutic target for RCC.

**Graphic abstract:**

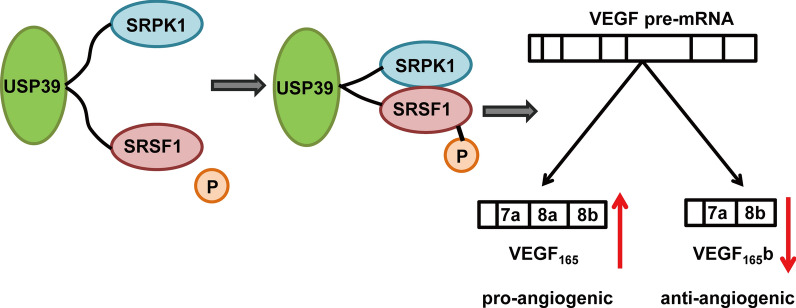

**Supplementary Information:**

The online version contains supplementary material available at 10.1186/s12935-021-02161-x.

## Introduction

Renal cell carcinoma (RCC) is one of the most lethal malignancies of the genitourinary system causing approximately 140,000 deaths each year worldwide [1, 2]. About 70% RCC patients were diagnosed with localized RCC in the early stage, which could be potentially cured by radical nephrectomy [3]. However, the other RCC patients may have developed metastasis at the initial diagnosis [4]. In addition, 20–40% RCC patients may experience recurrence or metastasis one or two years after the initial surgery with poor survival and prognosis [5]. Conventional therapies including radiotherapy or chemotherapy have limited and disappointing efficacy for advanced RCC patients [6]. Although the advent of targeted therapy including tyrosine kinase inhibitors (TKIs) has improved overall survival (OS) and progression-free survival (PFS) of patients with advanced RCC, drug resistance and rapid disease progression occurred frequently [7]. Therefore, it is an urgent task to gain novel insights into the mechanisms and therapeutic targets of RCC.

Accumulating studies have demonstrated that mutations or activities of RNA splicing-related factors participate in the development and progression of various malignant tumors [8, 9]. Ubiquitin-specific peptidase 39 (USP39), first found in yeast, is known as a type of protein associated with the assembly process of spliceosomal snRNP during pre-mRNA maturation [10, 11]. The molecular structure of USP39 consists of three domains: RS-like domain of N-terminal, ubiquitin binding domain of zinc finger protein (ZnF-UBP) in the middle, and ubiquitin-specific protease (USP) domain of C-terminal, without the activity of ubiquitin enzyme [12, 13]. It was reported that USP39 upregualtion was correlated with the development of medullary thyroid carcinoma (MTC) and human hepatocellular carcinoma (HCC) [14, 15]. It was found in our previous study that USP39 knockdown could inhibit the abnormal proliferation of prostate cancer cells by inhibiting the splicing maturation and transcriptional prolongation of EGFR mRNA [16]. Other studies have also reported that USP39 serves as the pro-tumor factor in many malignant tumors such as gastric cancer [17], osteosarcoma [18], lung cancer [19], glioma [20], and breast cancer [21]. In addition, USP39 knockdown was found to inhibit RCC progression through blocking Akt/ERK pathways [22]. However, the role of USP39 on splicing complex regulation in RCC progression remains unclear.

Tumor neovascularization originates from the abnormal pathological process of angiogenesis due to the imbalance between promoters and inhibitors [23]. Overexpression of vascular endothelial growth factor (VEGF), especially VEGF-A, has been documented as a stimulator of tumor angiogenesis [24]. VEGF can produce different isoforms through mRNA splicing including VEGFA-_165b_ [25], which is believed to be an anti-angiogenetic factor and downregulated in RCC, prostate cancer, colorectal cancer and melanoma [26–29]. It has been reported that Serine/Arginine-Rich Protein Specific Kinase 1 (SRPK1) could phosphorylate Serine/Arginine-Rich Splicing Factor 1 (SRSF1) to promote VEGF-A splicing to generate VEGF-_A165_ (pro-angiogenesis) and VEGF-A_165b_ (anti-angiogenesis) [30–32]. It is therefore hypothesized that VEGF-_165b_ or other anti-angiogenic splicing isoforms may become a promising therapeutic target or mediator of malignant tumors. However, the biological function and underlying mechanisms of VEGF splicing in RCC need to be elucidated.

The aim of the present study was to investigate the role of USP39 in RCC cell proliferation, malignant progression and angiogenesis and the potential mechanism of VEGF-A alternative splicing, in an attempt to gain deeper insights into the molecular mechanism underlying the development of RCC and provide new clues for exploring molecular targeted therapies of RCC.

## Materials and methods

### Bioinformatics data analysis

A series of survival data, expression level data, clinical characteristics were obtained from ONCOMINE database (www.oncomine.org) by using the following search terms: ‘USP39’, ‘Cancer vs. Normal Analysis’, ‘Kidney Cancer’ and ‘mRNA’. Two datasets (**Gumz Renal dataset and Jones Renal dataset**) including RCC vs. normal kidney tissues were used to analyze the expression of USP39 in RCC and normal kidney tissues [33]. One dataset (Zhao Renal dataset) with 176 RCC tissues was used to explore the effect of USP39 on survival analysis [34]. All data are reported Log2 Median-Centered intensity in the Oncomine database.

### Cell culture and treatments

A498, 769P, 786-O, ACHN, Caki-1, 293 T and human umbilical vein endothelial (HUVEC) cells were purchased from the Cell Bank of Shanghai Academy of Life Sciences, the Chinese Academy of Sciences. Cells were cultured with 1640 or DMEM + 10% fetal bovine serum (FBS) + 1% penicillin at 37 °C and 5% CO2.

Recombinant plasmids pcDNA3-USP39 were constructed to overexpress USP39 in our laboratory. The two truncated forms of USP39, one with amino acids (AA) _1–100_ (containing the RS-like domain, USP39 _(1–100)_) and the other with AA _101–565_ (containing the ZnF, UCH1, and UCH2 domains, USP39 _(101–565)_), were acquired from the Key Laboratory of Cell Differentiation and Apoptosis of the National Ministry of Education (Shanghai Jiaotong University School of Medicine, Shanghai, China)[35]. Lentiviral USP39 knockdown plasmids (Lv-shUSP39, ShRNA sequences: 5′-GATTTGGAAGAGGCGAGATAA-3′) were prepared by Hollybio Biotechnology Company (Shanghai, China). Empty plasmids (Con) and Lentiviral vector with nonspecific shRNA (Lv-shCon) were used as controls. Lentiviruses were constructed in 293 T cells according to the manufacturer’s method. The knockdown and overexpression efficiency was assessed by RT-PCR and Western blot. USP39 knockdown or overexpression plasmids were transfected into 786-O and ACHN cells and the HUVEC received cell supernatants of 786-O and ACHN cells with knockdown or overexpression USP39.

### MTT assay

Cells with stable knockdown of USP39 were cultured in a 96-well plate at 2000 cells/well for Day 1, 2, 3, 4 and 5. At each time point, cells were incubated with 3-(4, 5-dimethylthiazol-2-yl)-2,5-diphenyltetrazolium bromide (MTT) solution for 4 h at 37 °C and was terminated by acidic isopropanol solution. Half an hour later, cell viability was measured by 595 nm absorbance to acquire OD values. OD values of each day were used to draw the MTT growth curve. Each experiment was performed in triplicate.

### Plate colony formation assay

Cells with stable knockdown of USP39 were cultured in a 6-well plate at 400 cells/well at 37 °C for 14 days with the culture medium replaced at 3-day intervals. Cell colonies were fixed with 4% paraformaldehyde for 30 min and then stained under GIEMSA for 20 min. Each cell colony was counted under a light microscope and photographed with a digital camera.

### Flow cytometric assay and cell cycle detection

According to manufacturer’s instructions, cells were centrifuged, resuspended with PBS, fixed by addition of proof ethanol to a final ratio at 66%, incubated on ice for 15 min, resuspended in a working solution with 500 µl Propidium Iodide (PI) buffer, 25 µl PI (20×) and 5 µl RNase A (50×), and incubated again at 37 °C for 40 min. Flow cytometric analysis was performed using a flow cytometer (BD Biosciences, USA). Red fluorescence was detected at the excitation wavelength 488 nm, and the laser emission was detected at the same time.

### Tubule formation assay

After 24-h serum-free culture, cell supernatants of 786-O and ACHN cells with knockdown or overexpression USP39 were collected. After addition of 50 μl Matrigel solution to each well of the 96-well plate, cells were incubated at 37 °C for 1 h. HUVECs were resuspended with the collected cell supernatant or serum-free 1640 medium at 1 × 10^5^ cells/ml after serum-starvation overnight. The resuspended HUVECs (100 μl) were added to the Matrigel-coated wells and incubated for 8 h at 37 °C. Subsequently, HUVECs were imaged with an inverted fluorescence microscope (CKX41, Olympus, Japan). ImageJ was applied to analyze the number of meshes, the number of branches, and the tube length.

### Quantitative real-time PCR (RT-qPCR)

Total RNA was extracted with Trizol reagents (Invitrogen) and cDNA was obtained using First-Strand cDNA Synthesis Kit (Invitrogen). The resulting cDNA was subjected to RT-qPCR with the indicated primer sets. RT-qPCR analysis was conducted by Power SYBR Green PCR Master Mix (Applied Biosystems, Foster City, CA, USA). Relative gene expression was normalized to GAPDH with the 2^−ΔΔCT^ assay. The primer sequences were used: for USP39, 5′-GCCAGCAGAAGAAAAAGAGC-3′ (forward) and 5′-GCCATTGAACTTAGCCAGGA-3′ (reverse); for VEGF-A, 5′- GCACATAGGAGAGATGAGCTTCC-3′ (forward) and 5′- CTCCGCTCTGAACAAGGCT-3′ (reverse) for β-actin, 5′-ATCGTGCGTGACATTAAGGAG-3′ (forward) and 5′-AGGAAGGAAGGCTGGAAGAG-3′ (reverse). Values were normalized to those of Actin.

### Western blot

Cells with stable overexpression or knockdown of USP39 were lysed using RIPA lysis buffer (Beyotime, China), and the protein concentration was measured by BCA assay (Beyotime). Samples were prepared in SDS sample loading buffer, and transferred to the PVDF membrane. The main antibodies of Western blot were anti-USP39 (Abcam), anti-SRSF1 (Abcam), anti-SRPK1 (Santa Cruz), anti-VEGFA_165b_ (R&D, MAB3045), and anti-VEGF-A (Abcam, ab214424). The membrane was blocked by 5% milk (nonfat) in TBS (0.05% Tween 20) at room temperature for 1 h, and incubated with the primary antibodies for 2 h and with HRP-conjugated IgG (rabbit). Western blotting detection system (Tanon) was applied to detect Chemiluminescence of the membrane.

### Affinity purification and mass spectrometry (AP-MS)

We performed AP-MS as previously described [35]. Briefly, cells were solubilized with lysis buffer (50 mM Tris pH 7.4, 150 mM NaCl, 2 mM EDTA, 10% glycerol, 0.5% NP-40, protease inhibitors, and phosphatase inhibitors) after harvest. Cell extracts were immunoprecipitated with beads. Immunoprecipitation was performed as described previously [35]. After chromatographic separation, the samples were analyzed by Q-Exactive mass spectrometer. The detection method was positive ion, the scanning range of the mother ion was 300–1800 m/z, and the resolution of the primary mass spectrometry was 70,000 at 200 m/z. AGC(Automatic Gain Control) target was 1e6, Maximum IT was 50 ms, and Dynamic exclusion time was 60.0 s. The mass charge ratio of polypeptides and polypeptide fragments was collected as follows: After each full scan, 20 fragments were collected (MS2 scan). MS2 Activation Type was HCD, Isolation window was 2 m/z, secondary mass spectrometry resolution was 17,500 at 200 m/z. Normalized Collision Energy was 30 eV; Underfill was 0.1%.

### Co-immunoprecipitation (CO-IP) analysis

Cells with stable overexpression or knockdown of USP39 were collected by RIPA buffer. Immunoprecipitation was conducted with anti-HA (Abcam) or anti-SRPK1 (Santa Cruz) or anti-Pan-phospho-SR (Santa Cruz). By incubation with protein A agarose (Santa Cruz), the antibodies were removed. Proteins were prepared and separated by 10% SDS-PAGE. The interaction between USP39 and SRPK1/SRSF1 was analyzed by Western blot using anti-Flag tag (Abcam) or anti-SRSF1 (Abcam).

### Statistical analysis

All data were analyzed by SPSS version 22.0 (IBM corporation) and processed by GraphPad Prism 8.0. Independent sample *t*-test was used for comparison between groups. P < 0.05 was considered statistically significant.

## Results

### USP39 is highly expressed in clear cell renal cell carcinoma (ccRCC) and negatively correlated with survival of RCC patients

**Fig. 1:**
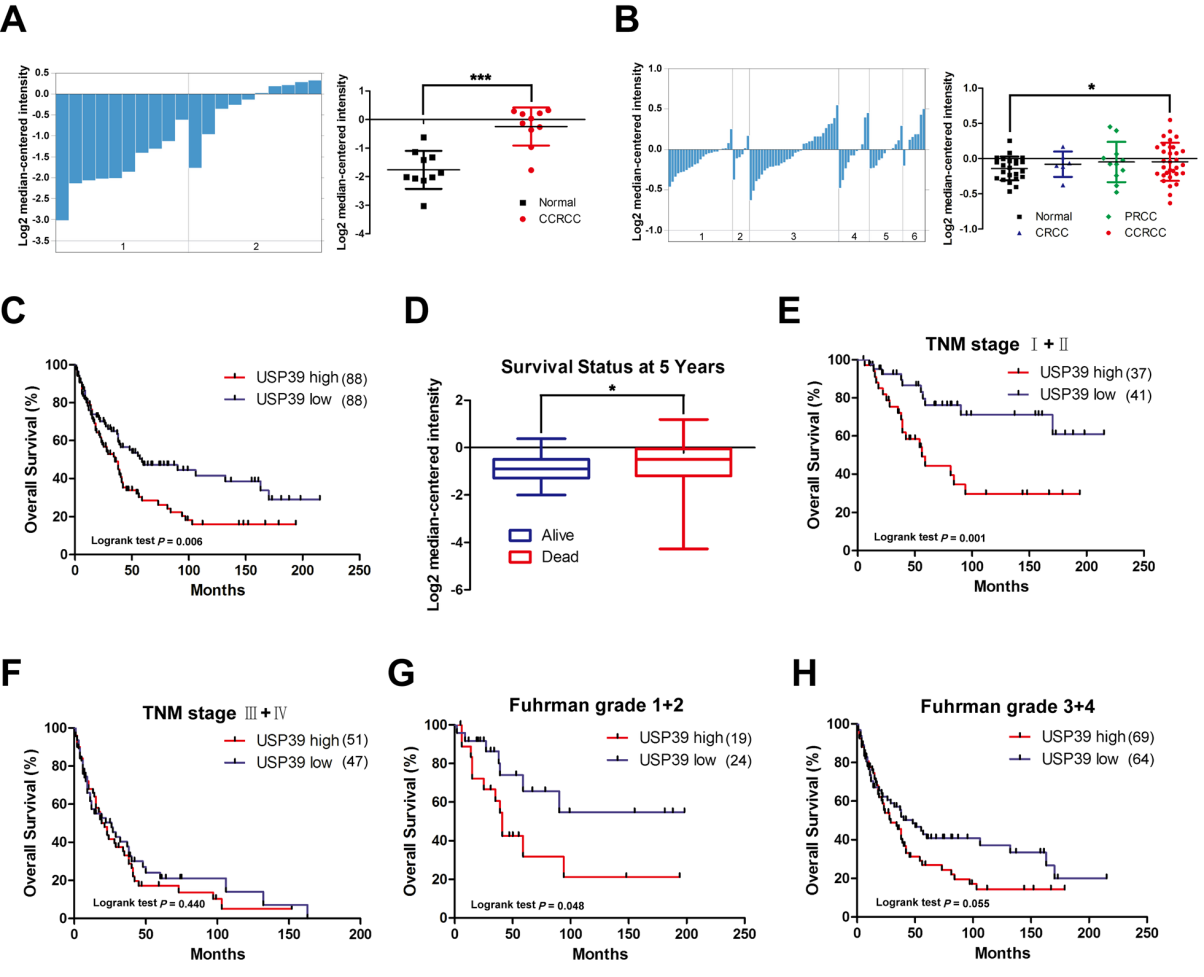
The value of USP39 expression in renal cell carcinoma by ONCOMINE gene database analysis. **A** The expression of USP39 in renal clear cell carcinoma was significantly higher than that in normal renal tissue. **B** USP39 expression was associated with the malignant degree of kidney cancer. **C** Patients with high expression of USP39 had poor survival and prognosis. **D** The expression level of USP39 in patients who died at 5 years and those who survived. **E**, **F** The overall survival of patients with high or low expression of USP39 in different TNM stages. **G**, **H** Overall survival of patients with high or low expression of USP39 in different Fuhrman grades. *CCRCC* renal clear cell carcinoma, *CRCC* color renal cell carcinoma, *PRCC* papillary renal cell carcinoma. *P < 0.05; ***P < 0.001 versus normal renal tissue. *P < 0.05 versus deaths at 5 years

To confirm the correlation between the expression level of USP39 in RCC and the prognosis of RCC patients, we retrieved the expression level of USP39 in all types of RCC and normal renal tissues through ONCOMINE database. The expression of USP39 in RCC tissue was significantly higher than that in normal renal tissue (P < 0.001) (Fig. 1A). In addition, the expression level of USP39 was associated with the malignant degree of RCC, with the highest USP39 expression in ccRCC vs*.* normal renal tissue (P < 0.05) (Fig. 1C).

We further investigated the survival data in the zhao database of ONCOMINE. As showed in Table1, patients with high expression of USP39 had a significantly lower survival rate (P < 0.01) and a shorter median survival duration as compared with those in patients with low expression of USP39 (P < 0.01), indicating that the expression level of USP39 was negatively correlated with the survival rate of RCC patients. Patients with high expression of USP39 had poorer survival prognosis (P = 0.006) (Fig. 1C), and the expression level of USP39 in patients who died at 5 years was significantly higher than that in patients who survived (P < 0.05) (Fig. 1D). In addition, we conducted a comparative analysis based on the stages of renal cancer patients and the expression level of USP39, and found that high expression of USP39 predicted poor survival prognosis in patients with a low TNM stage, while a high TNM stage showed no statistical difference in USP39 expression (P = 0.001, P = 0.440) (Fig. 1E, F). Concerning the pathological grade, USP39 predicted poor survival prognosis in patients with low Fuhrman grade, while patients with high Fuhrman grade showed no statistical difference in USP39 expression (Fig. 1G, H).

**Table 1:** USP39 expression and patient characteristics of Zhao Renal dataset

	No. Pts	No. USP39		*p* Value
		Low	High	
Overall: n (%)	176 (100.0)	88 (50.0)	88 (50.0)	0.286^a^
Male	101 (57.4)	47 (26.7)	54 (30.7)	
Female	75 (42.6)	41 (23.3)	34 (19.3)	
Mean patient age, y (range)	65.2 (34–85)	63.7(42–85)	66.6 (34–85)	< 0.000^b^
WHO performance status: n (%)				0.146^a^
0	65 (36.9)	29 (16.5)	36 (20.5)	
1	63 (35.8)	35 (19.9)	28 (15.9)	
2	37 (21.0)	16 (9.1)	21 (11.9)	
3	10 (5.7)	8 (4.5)	2 (1.1)	
4	1 (0.6)	0 (0.0)	1 (0.6)	
TNM stage: n (%)				0.919^a^
I	49 (27.8)	25 (14.2)	24 (13.6)	
II	29 (16.5)	16 (9.1)	13 (7.4)	
III	40 (22.7)	19 (10.8)	21 (11.9)	
IV	58 (33.0)	28 (15.9)	30 (17.0)	
Fuhrman grade: n (%)				0.726^a^
1	9 (5.1)	6 (3.4)	3 (1.7)	
2	34 (19.3)	18 (10.2)	16 (9.1)	
3	93 (52.8)	45 (25.6)	48 (27.3)	
4	40 (22.7)	19 (10.8)	21 (11.9)	
Patient outcome: n (%)				0.008^a^
Died	111 (63.1)	47 (21.6)	64 (27.3)	
Alive	65 (36.9)	41 (4.0)	24 (1.1)	
Median OS, mo (95% CI)	39 (33.4–44.6)	59 (14.1–103.9)	35 (23.0–47.0)	0.006^c^

Analysis of USP39 in the ONCOMINE gene database. The expression level of USP39 was positively correlated with the survival rate of renal cancer patients

^a^Chi-square test

^b^Wilcoxon rank sum test

^c^Log rank test

### USP39 expression was an independent risk factor for survival of RCC patients

**Fig. 2:**
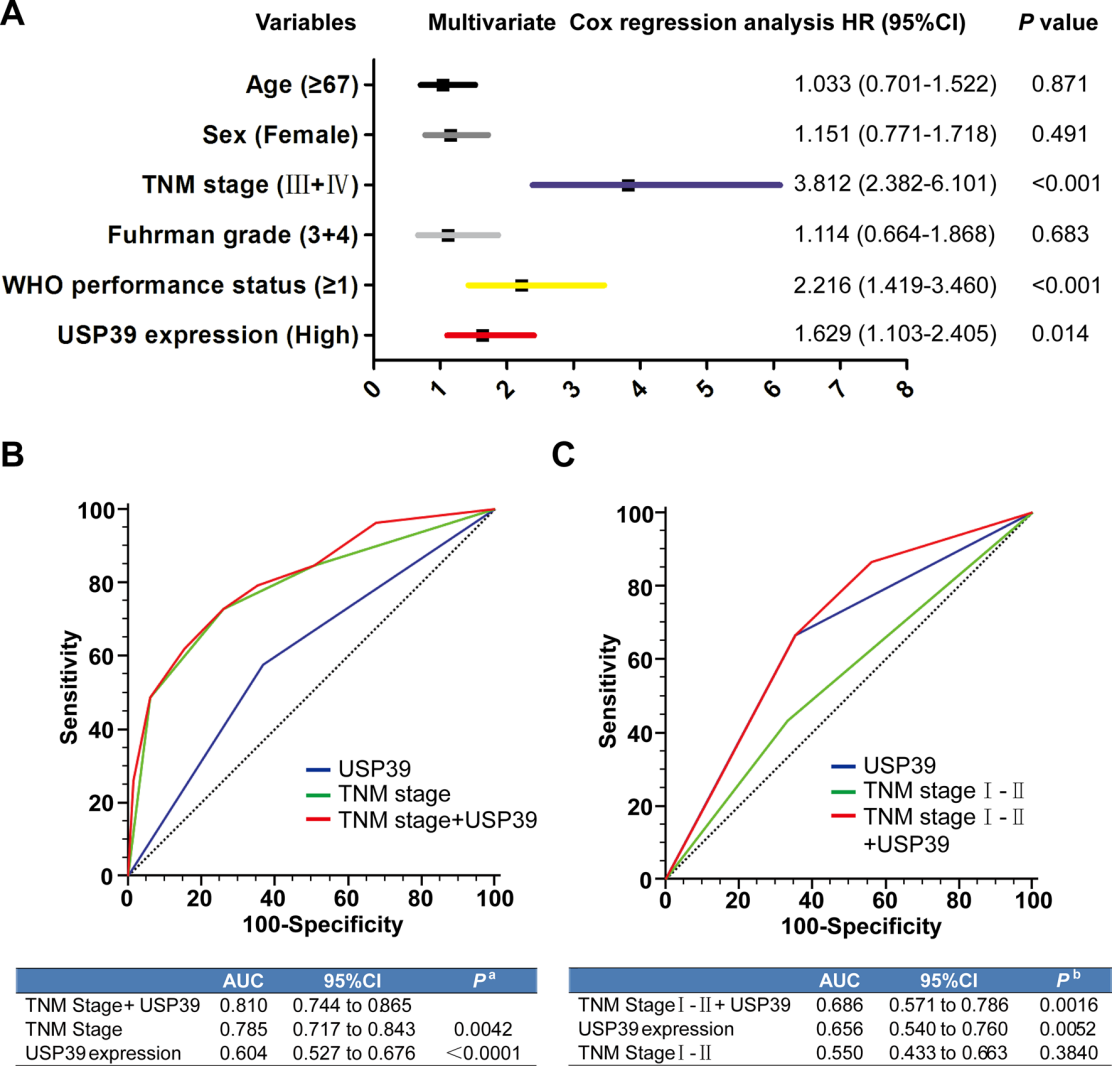
The expression of TNM combined with USP39 in patients with low stage can significantly predict the survival and prognosis of patients. **A** Forest plot showed that the level of USP39 expression is an independent risk factor for overall survival in renal cancer patients. **B** ROC curve analysis for predictive the ability of single indicator TNM, single indicator TNM and combined TNM and USP39. Blue line: USP39; Green line: TNM stage; Red line: TNM stage + USP39. **C** For patients with low TNM stage, ROC curve analysis for the predictive ability of the expression level of USP39, TNM stage, and combined indicators. Blue line: USP39; Green line: TNM stage I − II; Red line: TNM stage I − II + USP39

Next, we performed univariate and multivariate analyses to determine whether the expression of USP39 was an independent risk factor for survival of RCC patients. It was found that USP39 expression, TNM stage and the WHO performance status were independent risk factors for OS of RCC patients (Table 2, Fig. 2A). Considering that the high expression of USP39 predicted poor prognosis in patients with low TNM stage and TNM stage is currently recognized as an indicator for predicting survival and prognosis of patients with renal cell carcinoma, we hypothesized whether TNM stage combined with USP39 expression could improve the ability to predict prognosis in RCC patients. We used ROC curve to analyze the predictive ability of single index TNM stage, USP39 expression and combined indexes. The results suggested that combined TNM stage and USP39 expression had a higher predictive ability than a single index (P < 0.0001) (Fig. 2B). For patients with low TNM stage, the predictive ability of USP39 expression was stronger than that of TNM stage, and the combination of the two indexes could significantly improve the ability of predicting survival of RCC patients (P < 0.001) (Fig. 2C).

**Table 2:** Univariate and multivariate cox regression analysis of OS of Zhao renal dataset

	Univariate cox regression		Multivariate cox regression	
	HR (95% CI)	*p* value	HR (95% CI)	*p* value
Age^a^, years		0.125		0.871
< 67	1.00 (referent)		1.00 (referent)	
≥ 67	1.344 (0.921–1.959)		1.033 (0.701–1.522)	
Sex		0.736		0.491
Male	1.00 (referent)		1.00 (referent)	
Female	0.937 (0.642–1.367)		1.151 (0.771–1.718)	
TNM stage		0.000		0.000
I + II	1.00 (referent)		1.00 (referent)	
III + IV	4.279 (2.771–6.607)		3.812 (2.382–6.101)	
Fuhrman grade		0.013		0.683
1 + 2	1.00 (referent)		1.00 (referent)	
3 + 4	1.866 (1.138–3.060)		1.114 (0.664–1.868)	
WHO performance status		0.000		0.000
0	1.00 (referent)		1.00 (referent)	
1 or greater	2.460 (1.608–3.763)		2.216 (1.419–3.460)	
USP39 expression		0.007		0.014
Low	1.00 (referent)		1.00 (referent)	
High	1.680 (1.150–2.455)		1.629 (1.103–2.405)	

Univariate and multivariate analyses showed that the level of USP39 expression was an independent risk factor for overall survival in renal cancer patients

*OS* overall survival

^a^Divided at median

### USP39 knockdown inhibits proliferation and colony formation by inducing S arrest in 786-O and ACHN cell lines

**Fig. 3:**
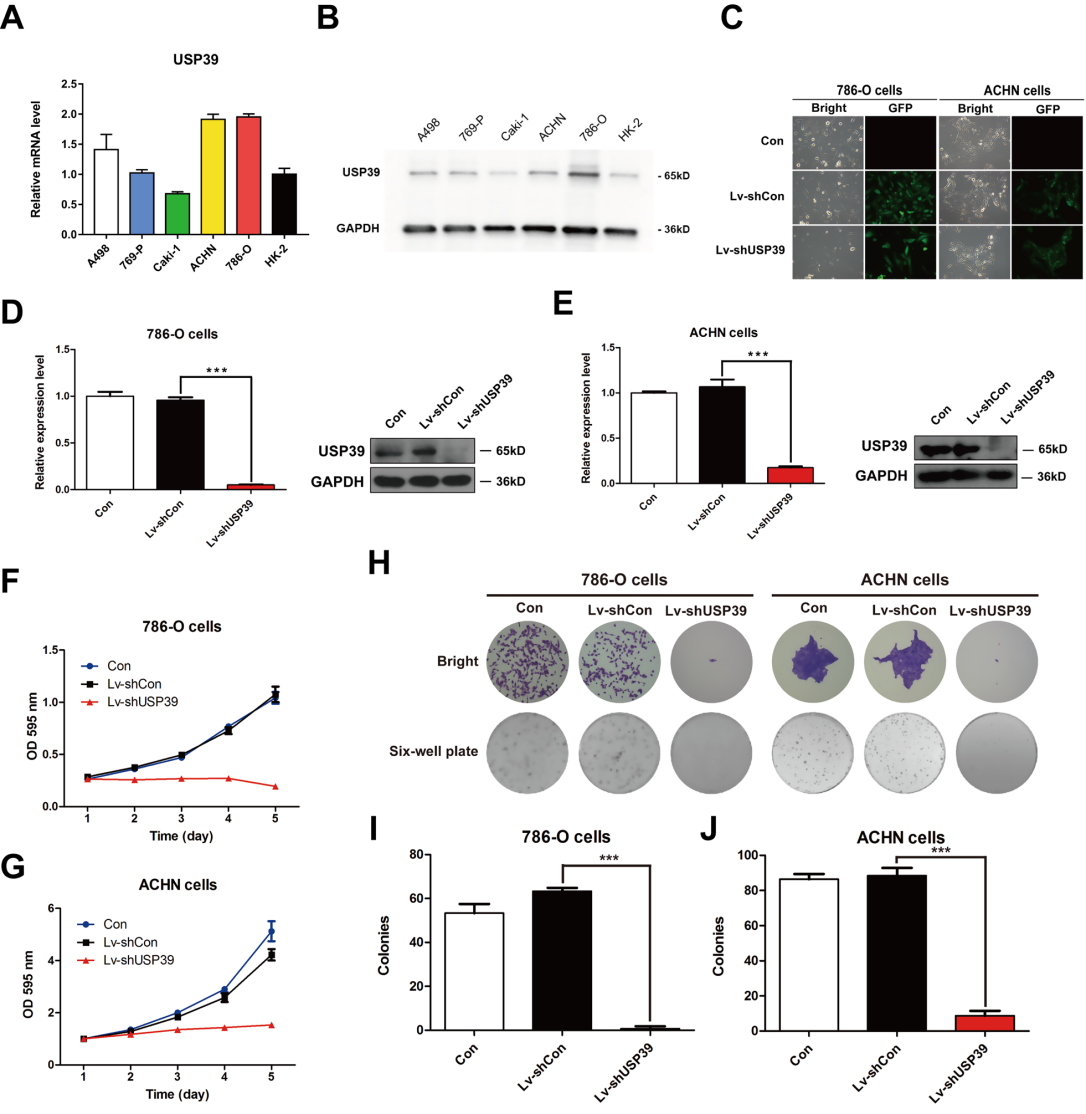
786-O and ACHN cell proliferation after USP39 knockdown. **A**, **B** The mRNA and protein expression levels of USP39 in renal cell carcinoma cell lines A498, 769-P, Caki-1, ACHN, 786-O. **C** Fluorescence expression of 786-O and ACHN cells after lentivirus infection. **D**, **E** The expression of USP39 after lentivirus infection as shown by Western blot assay and Real-time fluorescence quantitative PCR. **F**, **G** MTT growth curves of 786-O and ACHN cells after USP39 knockdown. **H**–**J** The cloning ability of 786-O and ACHN cells after USP39 knockdown. ***P < 0.001 versus Lv + shCon

We chose 786-O and ACHN with relatively high expression of USP39 as candidate target cell lines by comparing the expression of USP39 in five RCC cell lines and normal renal cell line (Fig. 3A, B). Then, Lentivirus-introduced shRNA (Lv-shUSP39) was used to silence USP39 expression in 786-O and ACHN cells (Fig. 3C). Western blotting and qRT-PCR were used to verify the efficiency of USP39 silencing. The results showed that the protein and mRNA expression levels of USP39 after knockdown were significantly lower than those of the control group (P < 0.001). Thus, 786-O and ACHN cell lines with USP39 knockdown were successfully prepared (Fig. 3D, E).

**Fig. 4:**
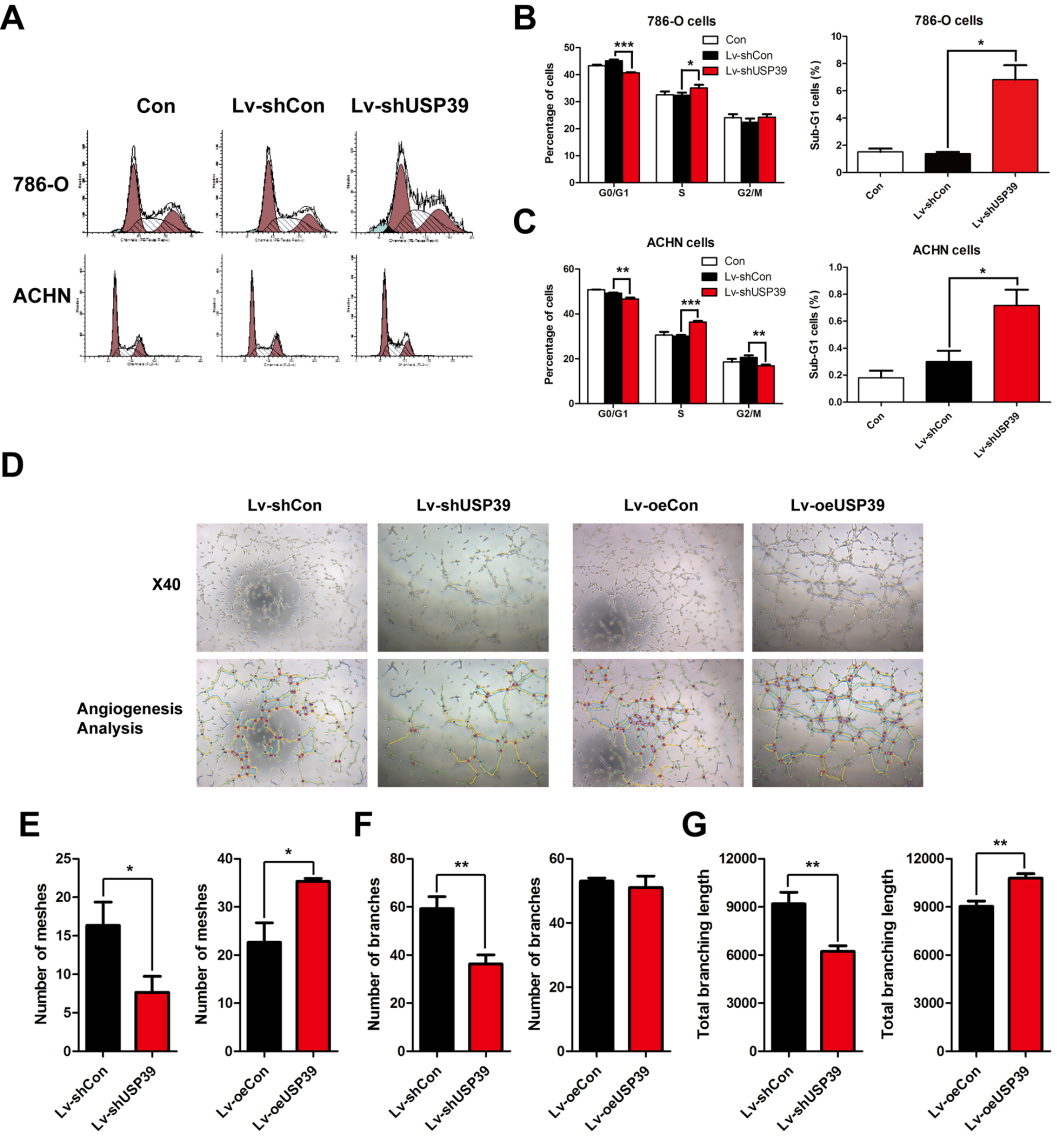
Changes in cell cycle of 786-O and ACHN cells after USP39 knockdown and the effect of USP39 on angiogenesis. **A** The S-phase peaks and subG1 phase peaks of 786-O and ACHN cells were significantly upregulated. **B**, **C** The proportion of S-phase cells, subG1 phase cells and G0/G1 phase cells in 786-O and ACHN cells after USP39 knockdown. **D** Tubular formation and analysis after USP39 knockdown or overexpression. **E**–**G** Tubular formation and tubule branching analysis of HUVECs after USP39 knockdown or overexpression. *P < 0.05; **P < 0.01 versus Lv + shCon or Lv + oeCon. *P < 0.05, **P < 0.01, ***P < 0.001 versus Lv + shCon

To explore the change of proliferation after USP39 knockdown in 786-O and ACHN cells, we applied MTT assay and colony formation assay. As shown in Fig. 3, the MTT growth curve indicated that the cell proliferation was significantly inhibited by USP39 silencing compared with the control group (Fig. 3F, G). And the results of cell colony formation assay showed that the number of the clones formed after knockdown of USP39 was significantly lower than that in the control group, and had statistical significance (P < 0.001) (Fig. 3H–J). The results of MTT assay and colony formation assay showed that knockdown of USP39 could significantly inhibit the malignant proliferation of RCC cells. Moreover, we performed FACS to analyze cell cycle distribution and evaluate the potential reason for the inhibition of proliferation. The results revealed that S arrest occurred due to USP39 silencing (P < 0.05) (Fig. 4A–C). These findings demonstrated that USP39 served as a strong pro-tumor factor in the malignant proliferation of RCC.

### Knockdown or overexpression of USP39 either inhibits or promotes tubule formation in vascular endothelial cells

RCC malignant proliferation is known to be correlated with tumor angiogenesis [36]. To investigate the effect of USP39 on the angiogenesis of vascular endothelial cells, we carried out tubule formation assay after co-incubation with cell supernatants of 786-O and ACHN cells with knockdown or overexpression USP39. It was found that USP39 overexpression significantly enhanced the tubule formation ability of HUVEC (P < 0.05), and increased the number of branches formed (P < 0.01) (Fig. 4D–G). After knockdown of USP39, the tube formation ability of HUVEC was inhibited (P < 0.01) (Fig. 4D–G), and the number of branches was decreased correspondingly (P < 0.01) (Fig. 4D–G). These findings indicated that knockdown or overexpression of USP39 could either inhibit or promote angiogenesis of endothelial cells.

### The USP39 _(101–565)_ fragment plays a biological role by binding and regulating SRSF1 and SRPK1

**Fig. 5:**
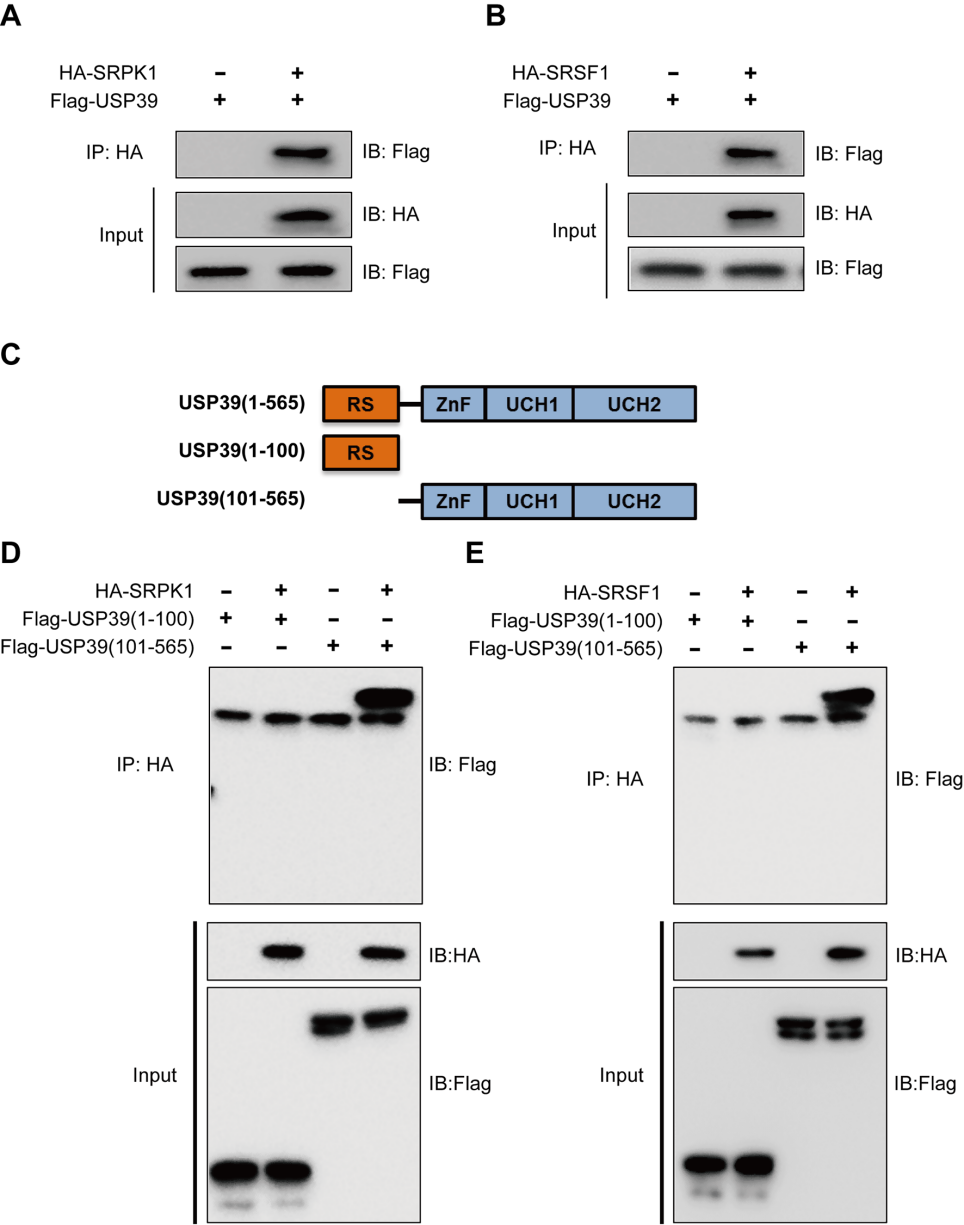
Mass spectrometry of interaction between USP39 and SRSF1 and SRPK1. **A** The plasmids were transfected into 293 T cells, and the interaction was verified in pairs for SRPK1. **B** The plasmids were transfected into 293 T cells, and the interaction was verified in pairs for SRSF1. **C** USP39 fragment plasmids were constructed. **D** SRPK1 interacted with the _(101–565)_ fragment of USP39. **E** SRSF1 interacted with the _(101–565)_ fragment of USP39

To explore the potential mechanism of USP39-mediated angiogenesis and malignant proliferation, affinity purification–mass spectrometry (AP-MS) was used to explore the interactions between USP39 and the potential target protein. It was found that USP39 combined with SRPK1 and SRSF1, which are regarded as key splicing factors involved in the alternative splicing of VEGF-A (Additional file 2: Table S1). To verify protein–protein interaction networks, Co-IP was carried out. As described by AP-MS, USP39 interacted with SRPK1 and SRSF1, respectively (Fig. 5A, B).

The molecular structure of USP39 consists of four domains: Arginine-serine-rich (RS)-like domain, ZnF domain, UCH1 domain, and UCH2 domain [10]. To determine which USP39 domain binds to SRPK1 and SRSF1, we received two truncated forms of USP39, including one RS-like domain (USP39 _1–100_) and one ZnF-UCH1-UCH2 complex domains (USP39 _101–565_) (Fig. 5C). Co-IP assay of USP39 truncated domain was performed with SRPK1 and SRSF1. The binding site of SRPK1 was located in the ZnF-UCH1-UCH2 complex domains (USP39 _101–565_), not in the RS-like domain (Fig. 5D). The same binding site was found in the interaction between USP39 and SRSF1 (Fig. 5E). These results indicated that USP39 acted through binding the USP39 _(101–565)_ domains to SRPK1/SRSF1.

**Fig. 6:**
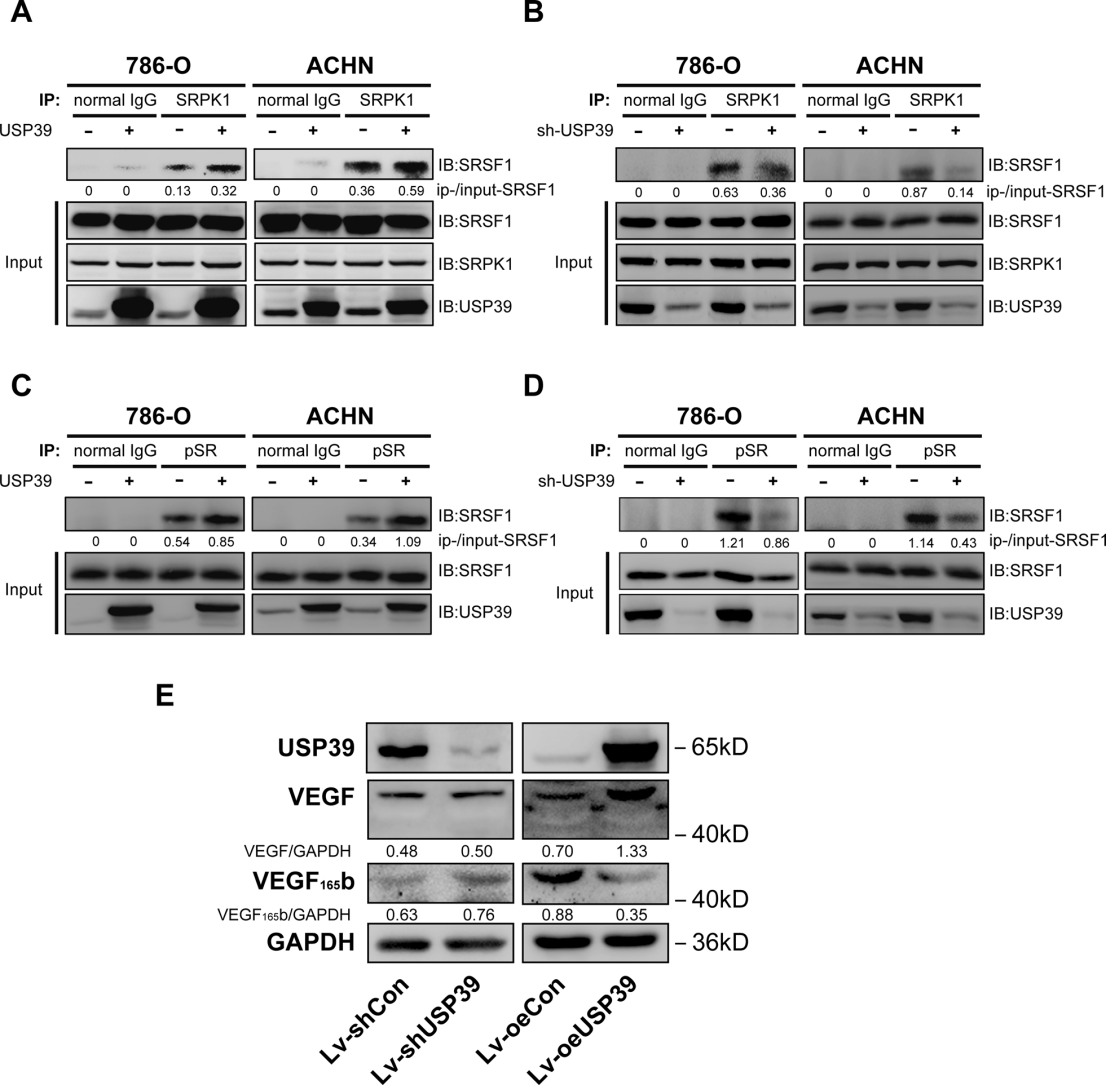
The expression level of USP39 affects the interaction between SRPK1 and SRSF1 and the effect of USP39 expression on VEGF-A variable splicing. **A** USP39 overexpression promoted the interaction between SRPK1 and SRSF1. **B** USP39 knockdown inhibited the interaction between SRPK1 and SRSF1. **C** USP39 overexpression promoted SRPK1 and SRPK1, and phosphorylation of SRSF1. **D** USP39 knockdown inhibited SRPK1 phosphorylation of SRSF1. **E** Total VEGF and VEGF-_165b_ expression after USP39 knockdown or overexpression. The densitometry quantification of the western blots under each blot

It has been reported that SRPK1 functions by regulating the phosphorylation of SRSF1 to promote VEGF-A alternative splicing [37]. To verify the regulatory effect of USP39 on SRPK1/SRSF1, we conducted Co-IP assay on 786-O and ACHN cells after knockdown and overexpression of USP39. It was found that overexpression of USP39 could enhance the interaction between SRPK1 and SRSF1 (Fig. 6A). On the contrary, knockdown of USP39 could weaken the interaction between SRPK1 and SRSF1 (Fig. 6B). In addition, there was stronger phosphorylation of SRSF1 in 786-O and ACHN cells with overexpression of USP39, and knockdown of USP39 in RCC cells could inhibit phosphorylation of SRSF1 (Fig. 6C, D). These findings indicated that USP39 affected the phosphorylation of SRSF1 by regulating the interaction between SRPK1 and SRSF1.

### Knockdown or overexpression of USP39 either upregulates or downregulates VEGF-A_165b_ expression

It was reported that SRPK1 directly acted on SRSF1 to promote the splicing of VEGF-A and enhance the production of VEGF-A165 and VEGF-A_165b_ [32]. Cancer cells with knockdown of SRPK1 increased the expression of VEGF-A_165b_ and reduced the expression of VEGF-A165 [30]. To verify the effect of USP39 on VEGF-A, the expression of VEGF-A and VEGF-A_165b_ was analyzed by 786-O cells with overexpression or knockdown of USP39. As shown in Fig. 6E, RCC cells with knockdown of USP39 upregulated the expression of VEGF-A_165b_. On the contrary, RCC cells with overexpression of USP39 downregulated the expression of VEGF-A_165b_. In addition, the RT-PCR results showed that USP39 affects the mRNA expression levels of VEGF 165 and VFGE 165b. USP39 knockdown significantly reduced the expression level of VEGF 165, but had no significant effect on overall VEGF-A (Additional file 1: Figure S1). It is worth noting that the splicing isomer of VEGF-A_165b_ could inhibit endothelial cell angiogenesis [29], which also supports the result of the above-mentioned tubule formation assay.

## Discussion

Kidney cancer ranks the top 10 common malignancies among all types of tumors, and RCC accounts for 85% of all kidney cancers [4] and 5% of epithelial malignant tumors diagnosed every year [38]. The median survival time of patients with metastatic RCC is only 12 months with a 5-year survival rate of less than 10% [39]. In addition, early diagnosis and prognostic judgement of RCC remain a challenge due to the lack of specific manifestations and a relatively high postoperative recurrence rate [40, 41]. It is reported in the literature that USP39 acts as a type of pro-tumor gene. Wen et al. [33] reported that overexpression of USP39 could promote the malignant proliferation of prostate cancer cells. Wang et al. [21] also found that USP39 was highly expressed in breast cancer cells, and down-regulation of USP39 could significantly reduce the proliferation and colony formation of breast cancer cells. However, these studies failed to explain the role of USP39 in early stage RCC (TNM stage I–II or Fuhrman grade 1–2). We demonstrated that the expression of USP39 was negatively correlated with the survival rate of RCC patients. High expression of USP39 could predict poor prognosis and played a significant role in patients with low TNM stage (TNM stage I–II) and low Fuhrman grade (Fuhrman grade 1–2). In addition, our univariate and multivariate analyses showed that the expression of USP39 was an independent risk factor for OS of RCC patients. The predictive value of TNM stage for early stage RCC was relatively poor, while combined with USP39 expression could significantly predict the survival and prognosis of RCC patients. In addition, knockdown of USP39 could significantly inhibit the malignant proliferation, cell colony formation and cell cycle blockage of RCC 786-O and ACHN cells, suggesting that USP39 is an important pro-oncogene in RCC, which is consistent with the study of *Xu* et al. in RCC [22].

Targeted therapy plays an anti-tumor role by reducing the size of primary tumors and metastatic sites. Among several targeted therapies, the research on VEGF-A antibodies has become a breakthrough in the treatment of patients with metastatic RCC, such as bevacizumab, which brings new hope for the limited efficacy of TKIs [42]. VEGF-A belongs to the family of platelet-derived growth factors, which is a critical endothelial cell-specific mitogen and vascular permeability inducing factor, stimulating tumor angiogenesis [43]. High expression of VEGF-A mRNA could be detected in almost all malignant tumors [44]. Clinical studies showed that individuals with highly expressed VEGF-A were associated with an increased RCC risk [45, 46]. A previous investigation demonstrated that RCC patients with VEGF-A-2578 genotype had poor prognosis, including a higher death risk, a larger tumor size, and a worse tumor grade, compared with patients carrying other genotypes [47]. In a study exploring the correlation between angiogenetic markers and RCC outcomes, VEGF-A was found positive in more tumors of immunohistochemistry results [48]. The VEGF-A gene contains eight exons, which are not present in the one VEGF-A at the same time [28]. Instead, the eight exons are recombined by alternative splicing of pre-mRNA to produce different VEGF-A subtypes, which determine their structure, function and affinity to the receptor [49]. Like previous analysis, traditional VEGF-A subtypes are angiogenic, including exon 1–5, 6a, 6b, 7a, 7b and 8a, which are usually identified as VEGF-A_xxx_, xxx for the number of amino acids [50]. In 2002 and 2004, *Bates *et al. identified another VEGF-A_xxx_ subtype, whose C-terminal exon formed an alternative open reading frame containing six amino acids because of distal splicing, usually called VEGF-A_xxxb_ [25, 28]. The pro-angiogenic VEGF-A165a would be overexpressed and predominated over the VEGF-A_165b_ under cancer conditions. VEGF-A_xxx_ usually promotes angiogenesis, while VEGF-A_xxxb_ is just the opposite [51, 52]. Although the mechanism of VEGF-A_xxxb_ inhibiting angiogenesis has not been elucidated, studies in recent years have revealed that VEGF-A_165b_ can bind to VEGFR-1 and VEGFR-2, but only slightly initiate receptor signal to induce tyrosine phosphorylation, thus reducing angiogenesis [26, 53]. Therefore, some studies reported that the VEGF-A level was not related to RCC risk or outcomes, which potentially resulted from the role of VEGF-A_xxxb_ [54].

SRSFs are involved in splicing regulation and promoting U1 and U2 snRNP binding to splicing sites, whose activities are influenced by SRPKs [55, 56]. SRPK1 is the first identified protein kinase of SR [57]. SRPK1 and other SR protein kinases can phosphorylate SR proteins to facilitate spliceosome assembly [58]. The splicing of VEGF-A exon 8 has been widely studied, and several pathways are found to be associated with VEGF-A_xxxb_ formation. On one hand, growth factors stimulate SRSF1 phosphorylation via SRPK1, thereby allowing SRSF1 to be transported to the nucleus and bind to the proximal splicing site, leading to VEGF-A_xxxa_ isoform formation [59]. On the other hand, downregulation of SRSF1 and SRPK1 switches the splicing of VEGF-A mRNA to generate more VEGF-A_xxxb_ [32]. Upregulation of SRPK1 could lead to the pro-angiogenic isoform of VEGF overexpression and promote disease progression, resulting in poor outcomes [30, 60]. Tumor cells attempt to kidnap VEGF-A165a and expel VEGFA-_165b_ so that they could promote cancer development [59]. This critical process might arise form VEGF splicing regulation. And the inhibition of SRPK1 has been demonstrated to attenuate angiogenesis by altering VEGFA-165a to VEGFA-_165b_ in cancer and kidney-related study [32]. *Beatrice *et al. [61] reported that SRPK1 directly acted on SRSF1 to promote VEGF-A splicing to form VEGF-A165 and VEGF-A165b isomers in non-small lung cancer cells, and down-regulation of SRPK1 increased VEGF-A165b expression, while the selective SRPK1 inhibitors brought the anti-angiogenesis effect. In addition, SRPK1 binding SRSF1 has a positive effect on VEGF-A165a alternative splicing and a negative effect on VEGF-A_165b_ alternative splicing [62]. All these findings indicate a potential disease therapy, such as SRPKs or SRSFs inhibitor and their interaction blockers [63]. In our study, we also demonstrated that SRPK1 and SRSF1 were involved in the production of alternative splicing of VEGF-A_165b_ in RCC cells and promoted cancer progression, which is consistent with the anti-angiogenic effect of VEGF-A_165b_ in previous studies [51–53].

USP39 is a member of the de-ubiquitin enzyme family, and the SR-related protein 65KD participates in splice assembly without protease activity or ubiquitin activity [10], suggesting that USP39 may act as a mRNA splicing factor. *Makarova *et al. found that USP39 played a regulatory role in pre-mRNA maturation through U4/U6. U5 tri-snRNP and ZnF-UBP domains of USP39 were also found to be the key regions for recruiting and/or activating splice complexes [10, 64]. USP39 can promote the splicing maturation and normal function of oncogenes mRNA such as Aurora-B, RB1 and mdm-x by regulating splicing complex [12, 65, 66]. Interestingly, co-immunoprecipitation and mass spectrometry analysis in our study also revealed that USP39 could interact with SRPK1 and SRSF1 in RCC cells and participated in VEGFA mRNA splicing. In addition, USP39 overexpression could promote SRPK1 phosphorylation of SRSF1, while knockdown of USP39 worked oppositely, confirming that USP39 and SRPK1 play a role through direct binding rather than affecting their transcription or translation. This also suggests that USP39 has a negative effect on VEGF-A_165b_ alternative spliceosome, which fits the effect of SRPK1 on VEGF-A_165b_. Uniquely, USP39 bond with SRPK1 through fragments _(101–565)_ to promote the phosphorylation and interaction of SRSF1 by SRPK1. USPs are usually expected to be formed by an inactive ubiquitin-specific protease (iUSP) domain and a zinc finger ubiquitin binding domain (ZnF-UBP) [35, 64]. The ZnF of USP39 domain participates in the activities of neighboring domains dependent on ubiquitin but cannot bind ubiquitin itself because of the lack of zinc-binding sites [67]. This suggests that the biological role in mRNA alternative splicing requires additional mediation. A previous study reported that the RS-like domain (AA _1–100_) was the only binding truncation of SUMOylation instead of other domains crossing AA _101–565_ (ZnF domain, UCH1 domain, or UCH2 domain) in prostate cancer [35]. Previous concepts tend to believe that the N-terminal domain, like RS domains of SR proteins, has rich arginine/serine/glutamate, which is critical for recruiting TRI-snRNP into the pre-spliceosome [68]. Interestingly, our results showed the ZnF domain, UCH1 domain, or UCH2 domain became the binding site. This potentially originating from the iUSP domain cannot bind ubiquitin either, and the ZnF of USP39 could interact with the splicing independent on ubiquitin [64], which provides the opportunity for SRSF1 binding to USP39_(101–565)_, though further exploration is required to confirm the conclusion.

Firstly, one remaining limitation is how USP39 drives the changes in VEGF-A_165b_, which needs further research to elucidate the specific mechanisms. Secondly, we used data from outside China for the analysis, which may bring potential population bias, though the sources of cell lines were consistent with the datasets. Thus, this study comprised data from both outside and inside China and consistent conclusions were obtained. Thirdly, the Proximity Ligation Assay was not used in our work to prove USP39 promotes the interaction of SRPK1 with SRSF1 due to the obvious results of Co-IP.

In summary, we firstly demonstrated the value of USP39 in predicting survival, recurrence and metastasis in RCC patients, especially in those with low TNM stage, and that TNM stage combined with USP39 expression was superior to the single index. We have discovered a new regulatory network among USP39, SRPK1, SRSF1 and VEGF-A_165b_, which can promote the tumorigenesis and development of RCC. USP39 downregulation could inhibit RCC cell proliferation and progression, suggesting that USP39 may prove to be a potential target for inhibiting RCC. These findings may provide a theoretical basis for the development of new targets for the treatment of RCC.

## Supplementary Information

**Additional file 1** **:** **Figure S1.**

**Additional file 2** **:** **Table S1.**

## Data Availability

All primary data presented in this study are available from the corresponding author upon reasonable request.
