# Supplementary figures and images for: USP39 promotes malignant proliferation and angiogenesis of renal cell carcinoma by inhibiting VEGF-A165b alternative splicing via regulating SRSF1 and SRPK1
Source: Cancer Cell Int. 2021 Sep 20;21:486. doi: 10.1186/s12935-021-02161-x (PMC8454004; doi:10.1186/s12935-021-02161-x)

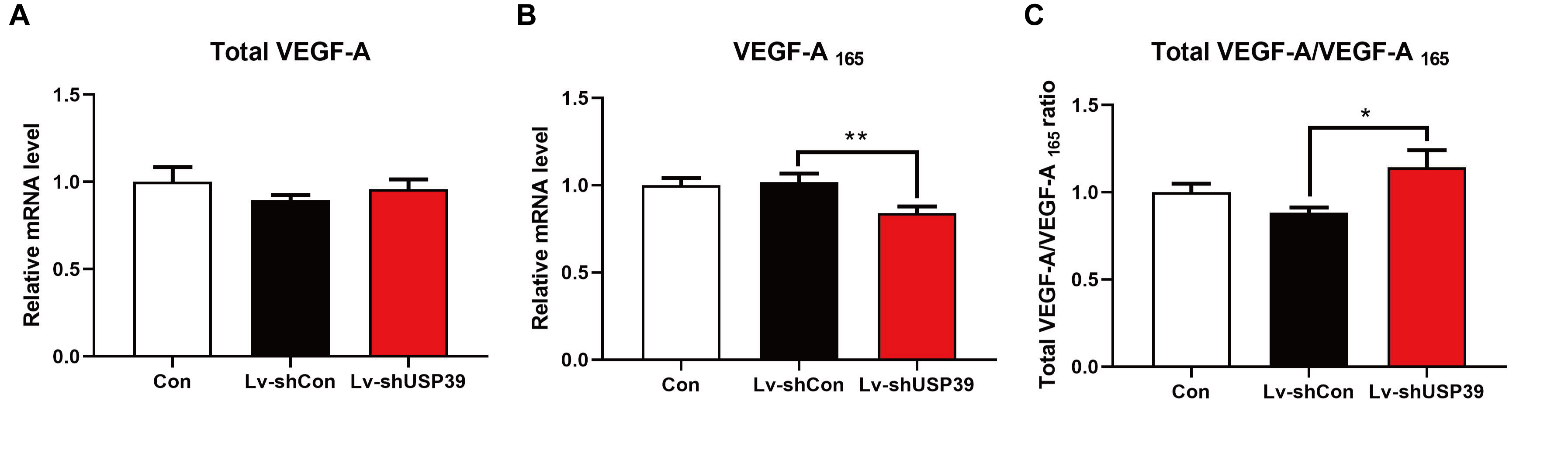

Supplement: Supplementary file 1 — Additional file 1 : Figure S1. [file 12935_2021_2161_MOESM1_ESM.jpg]
