# Supplementary material for: USP39 promotes malignant proliferation and angiogenesis of renal cell carcinoma by inhibiting VEGF-A165b alternative splicing via regulating SRSF1 and SRPK1
Source: Cancer Cell Int. 2021 Sep 20;21:486. doi: 10.1186/s12935-021-02161-x (PMC8454004; doi:10.1186/s12935-021-02161-x)
Supplement: Supplementary file 2 — Additional file 2 : Table S1. [file 12935_2021_2161_MOESM2_ESM.pdf]

Accession	Description	ΣCoverage	Σ# Proteins	Unique Pept	Σ# Peptide	Σ# PSMs	Score G91	Coverage G91	Peptides G9	# PSM G91	Score HY	Coverage HY	# Peptides HY	# PSM HY	# AAs	MW [kDa]	calc. pI
Q53GS9	otein 2 OS=Homo sapiens GN=USP	79.47	10	45	45	662	10476.50	79.47	45	600	770.53	48.32	18	62	565	65.3	8.91
P19338	mo sapiens GN=NCL PE=1 SV=3	35.07	9	14	30	86	1784.22	35.07	30	77	385.85	11.27	5	9	710	76.6	4.70
H7BY16	S=Homo sapiens GN=NCL PE=1 SV	53.20	1	1	17	58	1130.42	53.20	17	49	385.85	26.94	5	9	297	32.4	5.36
P01859	OS=Homo sapiens GN=IGHG2 PE	12.88	5	2	2	149	944.35	12.88	2	73	1304.00	10.12	1	76	326	35.9	7.59
A8K4Z4	n, large, P0 (RPLP0), transcript var	59.94	19	14	14	38	843.76	59.94	14	35	76.36	9.15	2	3	317	34.2	5.97
Q02878	5 OS=Homo sapiens GN=RPL6 PE=	45.49	12	16	16	44	803.05	45.49	16	41	43.55	11.11	2	3	288	32.7	10.58
O75643	Da helicase OS=Homo sapiens GN=	31.84	10	51	51	54	698.70	31.84	51	54					2136	244.4	6.06
P67809	protein 1 OS=Homo sapiens GN=Y	44.14	12	9	14	33	649.84	44.14	14	31	53.03	5.25	1	2	324	35.9	9.88
A0A087WTT1	S=Homo sapiens GN=PABPC1 PE=	43.68	31	13	21	36	586.63	43.68	21	35	46.34	4.60	1	1	522	58.5	9.26
P16403	mo sapiens GN=HIST1H1C PE=1 S	25.35	3	5	8	24	575.82	25.35	8	24					213	21.4	10.93
P62906	OS=Homo sapiens GN=RPL10A PE	49.77	3	12	12	34	571.10	49.77	12	34					217	24.8	9.94
P05387	P2 OS=Homo sapiens GN=RPLP2	79.13	2	9	9	18	569.90	79.13	9	18					115	11.7	4.54
P62888	OS=Homo sapiens GN=RPL30 PE	72.17	8	9	9	23	548.51	72.17	9	23					115	12.8	9.63
P30050	OS=Homo sapiens GN=RPL12 PE	60.00	4	7	7	23	546.56	60.00	7	22	64.88	9.09	1	1	165	17.8	9.42
P16989	OS=Homo sapiens GN=YBX3 PE=1	38.44	6	1	10	24	530.24	38.44	10	22	53.03	4.57	1	2	372	40.1	9.77
P11142	tein OS=Homo sapiens GN=HSPA8	47.99	28	22	25	38	527.16	47.99	25	38					646	70.9	5.52
A0A024RAV4	CRA_b OS=Homo sapiens GN=CSD	46.86	6	1	10	24	522.71	46.86	10	22	53.03	5.61	1	2	303	31.9	9.66
P08107	1B OS=Homo sapiens GN=HSPA1A	32.76	16	16	18	26	504.38	32.76	18	26					641	70.0	5.66
P62424	OS=Homo sapiens GN=RPL7A PE	54.14	4	18	18	30	499.00	54.14	18	29	37.93	9.02	1	1	266	30.0	10.61
P36578	4 OS=Homo sapiens GN=RPL4 PE=	43.56	10	16	16	28	461.91	43.56	16	28					427	47.7	11.06
B4DY09	tor 2 OS=Homo sapiens GN=ILF2	44.32	6	11	11	20	444.70	44.32	11	20					352	38.9	4.94
Q07955	or 1 OS=Homo sapiens GN=SRSF1	34.68	8	10	10	19	426.25	34.68	10	19					248	27.7	10.36
P26368	unit OS=Homo sapiens GN=U2AF2	40.63	3	14	14	30	418.34	40.63	14	28	31.96	2.74	1	2	475	53.5	9.09
Q6P2Q9	ctor 8 OS=Homo sapiens GN=PRP	25.52	8	46	46	48	400.78	25.52	46	48					2335	273.4	8.84
Q9NZI8	g protein 1 OS=Homo sapiens GN	26.69	2	11	13	16	392.47	26.69	13	16					577	63.4	9.20
B3KX19	Da U5 small nuclear ribonucleoprot	32.74	12	23	23	29	383.69	32.74	23	28	27.59	2.18	1	1	962	108.1	5.00
A8K590	ding factor 3, 90kDa (ILF3), transc	29.77	18	18	18	26	378.30	29.77	18	26					702	76.0	7.75
Q0QEW2	ent) OS=Homo sapiens GN=RPL18	44.51	9	7	7	14	371.10	44.51	7	13	34.18	7.93	1	1	164	18.7	11.81
B1ANR0	orm), isoform CRA_e OS=Homo sa	28.62	16	7	15	20	362.75	28.62	15	19	46.34	3.90	1	1	615	67.9	9.45
B3KPS3	ar to Tubulin alpha-ubiquitous cha	34.13	37	9	10	12	331.03	34.13	10	12					416	46.2	5.12
P46087	nethyltransferase OS=Homo sapier	27.71	8	17	17	18	317.60	27.71	17	18					812	89.2	9.23
D6R9P3	in A/B OS=Homo sapiens GN=HN	37.50	8	11	12	17	312.50	37.50	12	17					280	30.3	7.91
P11387	S=Homo sapiens GN=TOP1 PE=1	30.98	20	23	23	28	298.47	30.98	23	28					765	90.7	9.31
Q9NR30	OS=Homo sapiens GN=DDX21 PE=	28.10	9	19	19	26	286.12	28.10	19	26					783	87.3	9.28
P61313	OS=Homo sapiens GN=RPL15 PE	50.49	10	12	12	20	285.49	50.49	12	20					204	24.1	11.62
E4W6B6	nent) OS=Homo sapiens GN=RPL2	46.83	7	6	6	16	279.67	46.83	6	16					126	14.2	10.46
B3KY11	e ATP-dependent RNA helicase DD	30.38	8	22	22	22	272.55	30.38	22	22					800	93.2	9.50
O94906	6 OS=Homo sapiens GN=PRPF6 P	23.17	1	20	20	22	265.49	23.17	20	22					941	106.9	8.25
Q0VGD6	OS=Homo sapiens GN=HNRPR PE=	32.62	14	17	17	19	263.44	32.62	17	19					607	67.8	8.91
P62241	8 OS=Homo sapiens GN=RPS8 PE=	44.71	3	8	8	14	260.70	44.71	8	14					208	24.2	10.32
P18124	7 OS=Homo sapiens GN=RPL7 PE=	41.94	6	10	10	13	258.15	41.94	10	13					248	29.2	10.65
Q9HCE1	OS=Homo sapiens GN=MOV10 PE=	20.94	5	16	16	18	256.05	20.94	16	18					1003	113.6	8.82
Q9NW13	S=Homo sapiens GN=RBM28 PE=1	16.34	5	11	11	12	252.23	16.34	11	12					759	85.7	9.22
Q8TBW1	n (Fragment) OS=Homo sapiens P	41.71	15	14	14	17	245.51	41.71	14	17					374	42.7	9.99
J3KP15	Fragment) OS=Homo sapiens GN=	38.06	13	3	5	8	243.63	38.06	5	8					134	15.5	11.06
P62917	8 OS=Homo sapiens GN=RPL8 PE=	33.46	6	7	7	12	243.14	33.46	7	12					257	28.0	11.03
B4DZQ5	in kinase PRP4 homolog (EC 2.7.1	19.23	3	1	14	16	240.81	19.23	14	16					993	115.4	10.27
A0PJJ5	OS=Homo sapiens GN=SRP72 PE=	34.17	8	16	16	17	238.68	34.17	16	17					559	62.6	8.32
F8W6I7	ein A1 OS=Homo sapiens GN=HNR	22.80	16	5	5	5	236.62	22.80	5	5					307	33.1	9.13
Q13523	homolog OS=Homo sapiens GN=P	19.36	3	1	14	16	229.51	19.36	14	16					1007	116.9	10.26

Q92522	lomo sapiens GN=H1FX PE=1 SV=	28.17	1	6	6	8	228.71	28.17	6	8					213	22.5	10.76
A8K7N0	ribosomal protein L14, mRNA OS=	31.19	6	1	6	12	228.59	31.19	6	11	64.98	5.50	1	1	218	23.6	10.84
E7ETK0	)S=Homo sapiens GN=RPS24 PE=	29.77	3	4	4	9	228.16	29.77	4	9					131	15.2	10.89
P49207	OS=Homo sapiens GN=RPL34 PE	37.61	2	7	7	12	226.82	37.61	7	12					117	13.3	11.47
A8K644	form CRA_b OS=Homo sapiens GN	20.65	6	4	10	13	223.85	20.65	10	13					494	56.7	11.52
B7ZKK5	mo sapiens GN=EPO PE=2 SV=1	12.50	4	2	2	12	219.73	12.50	2	6	292.86	12.50	2	6	192	21.2	7.75
Q59GY3	variant (Fragment) OS=Homo sap	31.54	7	4	10	13	218.49	31.54	10	13					279	31.8	10.95
Q86Y74	)S=Homo sapiens GN=CROP PE=2	49.40	11	11	11	12	217.91	49.40	11	12					251	28.9	7.01
Q02543	OS=Homo sapiens GN=RPL18A PE	39.77	12	7	7	9	204.60	39.77	7	9					176	20.7	10.71
P68104	OS=Homo sapiens GN=EEF1A1 PE	16.88	37	6	6	12	202.77	16.88	6	9	96.27	6.28	1	3	462	50.1	9.01
Q6IPH7	mo sapiens GN=RPL14 PE=2 SV=	31.82	6	1	6	11	201.30	31.82	6	10	64.98	5.45	1	1	220	23.8	10.93
H0YA96	) (Fragment) OS=Homo sapiens GI	19.05	12	2	4	6	199.67	19.05	4	6					210	23.9	9.58
O76021	otein 1 OS=Homo sapiens GN=RSL	34.08	9	15	15	15	196.64	34.08	15	15					490	54.9	10.13
P06748	Homo sapiens GN=NPM1 PE=1 SV	28.23	17	5	5	7	190.53	28.23	5	7					294	32.6	4.78
Q9Y383	-like 2 OS=Homo sapiens GN=LUC	34.69	14	13	13	17	186.18	34.69	13	17					392	46.5	10.01
A8K9U6	e, antiviral 1 (ZC3HAV1), transcript	17.85	5	12	12	13	184.33	17.85	12	13					902	101.4	8.35
Q86YZ3	no sapiens GN=HRNR PE=1 SV=2	13.44	1	9	9	14	183.91	11.05	8	10	68.46	6.56	4	4	2850	282.2	10.04
B4DTA2	nucleoprotein D-like (HNRPDL), tra	14.02	5	2	4	6	183.24	14.02	4	6					271	30.2	8.72
Q8NE71	ember 1 OS=Homo sapiens GN=A	25.56	8	17	17	20	182.50	25.56	17	20					845	95.9	6.80
P19474	121 OS=Homo sapiens GN=TRIM2	37.26	2	14	14	22	171.63	31.79	12	13	111.26	22.11	9	9	475	54.1	6.38
Q9UHX1	JF60 OS=Homo sapiens GN=PUF6	20.04	7	8	8	9	166.76	20.04	8	9					559	59.8	5.29
B4DLM0	region-containing protein 2 OS=H	23.66	26	10	10	12	164.99	23.66	10	12					503	56.3	10.39
F6KPG5	:) OS=Homo sapiens PE=2 SV=1 -	8.89	14	6	6	14	161.50	8.89	5	8	178.39	6.15	4	6	585	66.5	6.04
A1L407	Homo sapiens GN=HIST1H1T PE=	18.36	3	2	5	10	160.91	18.36	5	10					207	22.0	11.72
P23396	OS=Homo sapiens GN=RPS3 PE=	36.21	17	7	7	7	156.57	36.21	7	7					243	26.7	9.66
Q9H6S0	YTHDC2 OS=Homo sapiens GN=Y	11.26	5	13	13	17	148.09	11.26	13	17					1430	160.1	8.40
P42696	S=Homo sapiens GN=RBM34 PE=1	26.28	4	10	10	10	146.14	26.28	10	10					430	48.5	10.11
G3V576	ns C1/C2 OS=Homo sapiens GN=H	27.71	32	6	6	6	145.58	27.71	6	6					231	25.2	9.82
P11021	ein OS=Homo sapiens GN=HSPA5	5.96	2	1	3	4	143.75	5.96	3	4					654	72.3	5.16
O00425	g protein 3 OS=Homo sapiens GN	16.41	1	6	8	9	143.46	16.41	8	9					579	63.7	8.87
K7EMA7	)S=Homo sapiens GN=RPL23A PE=	34.29	7	2	2	3	140.19	34.29	2	3					70	7.9	9.23
Q16629	or 7 OS=Homo sapiens GN=SRSF7	32.35	5	7	8	10	139.74	32.35	8	10					238	27.4	11.82
Q01081	unit OS=Homo sapiens GN=U2AF1	37.50	7	6	6	9	139.61	37.50	6	8	32.79	10.83	1	1	240	27.9	8.81
Q86UE4	omo sapiens GN=MTDH PE=1 SV=	9.62	4	5	5	5	137.47	9.62	5	5					582	63.8	9.32
Q9Y3U8	OS=Homo sapiens GN=RPL36 PE	34.29	5	4	4	6	136.03	34.29	4	6					105	12.2	11.59
Q99848	EBP2 OS=Homo sapiens GN=EBNA	31.70	4	7	7	7	135.88	31.70	7	7					306	34.8	10.10
P46777	OS=Homo sapiens GN=RPL5 PE=	25.93	6	7	7	9	135.52	25.93	7	9					297	34.3	9.72
A0A087WUT6	5B OS=Homo sapiens GN=EIF5B	9.34	7	10	10	10	133.10	9.34	10	10					1220	138.6	5.58
B7Z8F4	RNA helicase DHX8 (EC 3.6.1.-) O	13.89	7	12	12	12	131.56	13.89	12	12					1181	133.9	7.88
Q08211	A OS=Homo sapiens GN=DHX9 P	7.24	4	8	8	8	130.11	7.24	8	8					1270	140.9	6.84
P38159	nosome OS=Homo sapiens GN=RI	22.76	13	8	8	9	128.83	22.76	8	9					391	42.3	10.05
P07305	Homo sapiens GN=H1F0 PE=1 SV=	23.20	1	4	4	5	127.50	23.20	4	5					194	20.9	10.84
Q5VTL8	OS=Homo sapiens GN=PRPF38B P	15.75	3	7	7	7	126.49	15.75	7	7					546	64.4	10.54
P62979	27a OS=Homo sapiens GN=RPS27	31.41	4	4	4	5	125.59	31.41	4	5					156	18.0	9.64
Q7KYM9	mo sapiens GN=ORF PE=2 SV=1 -	16.14	9	8	8	8	124.96	16.14	8	8					570	59.9	8.41
A0A024RCU9	_e OS=Homo sapiens GN=SRPK1 P	29.74	13	11	12	13	122.44	29.74	12	13					548	61.9	5.69
A0A024R326	_a OS=Homo sapiens GN=RPL29 P	14.65	3	2	2	4	122.43	14.65	2	4					157	17.5	11.66
B5BUB5	OS=Homo sapiens GN=SSB PE=2	27.21	8	11	11	11	119.80	27.21	11	11					408	46.8	7.12
P51991	tein A3 OS=Homo sapiens GN=HN	30.95	5	9	9	11	117.50	30.95	9	11					378	39.6	9.01
A0JLQ5	)S=Homo sapiens GN=BXDC2 PE=	21.79	5	6	6	8	115.66	21.79	6	8					335	39.1	9.91
Q5JP53	-Homo sapiens GN=TUBB PE=1 SV	44.84	54	3	13	17	115.55	44.84	13	17					426	47.7	4.81

Q15050	ein homolog OS=Homo sapiens GN	21.10	1	6	6	6	113.60	21.10	6	6					365	41.2	10.70
A0A024R0J9	soform CRA_a OS=Homo sapiens	12.17	13	8	8	9	110.77	12.17	8	9					756	84.7	8.78
P84103	or 3 OS=Homo sapiens GN=SRSF	32.32	2	5	6	6	110.41	32.32	6	6					164	19.3	11.65
Q05BU6	S=Homo sapiens GN=SFRS11 PE=	27.64	12	5	5	8	109.93	27.64	5	8					246	24.8	6.34
Q8IYB3	otein 1 OS=Homo sapiens GN=SRI	21.35	6	16	16	16	108.67	21.35	16	16					904	102.3	11.84
A0A0A0MS14	=Homo sapiens GN=IGHV1-45 PE=	9.40	1	1	1	5	107.70	9.40	1	4	44.36	9.40	1	1	117	13.5	9.10
B4DJK0	or 5 OS=Homo sapiens GN=SRSF5	16.94	6	1	2	5	107.44	16.94	2	5					124	14.4	10.08
A0A0A0MRK1	OS=Homo sapiens GN=GPATCH4	32.70	8	9	9	10	106.84	32.70	9	10					370	42.0	9.31
H0YEQ8	ment) OS=Homo sapiens GN=PAE	47.32	1	1	4	5	106.13	47.32	4	5					112	12.6	7.96
A0A087WV29	omo sapiens GN=NAT10 PE=1 SV	6.71	2	4	4	4	104.94	6.71	4	4					834	93.5	5.88
Q1ED39	. OS=Homo sapiens GN=KNOP1 P	21.40	4	8	8	8	103.55	21.40	8	8					458	51.6	9.86
Q8N1H4	no sapiens transformer-2-beta (SFI	28.17	7	5	5	6	103.14	28.17	5	6					252	29.2	10.65
P15924	Homo sapiens GN=DSP PE=1 SV=	4.81	7	12	12	14	101.65	4.35	11	11	60.11	1.11	3	3	2871	331.6	6.81
B4DKS8	us nuclear ribonucleoprotein F OS=	12.43	3	2	3	3	97.56	12.43	3	3					338	37.2	6.05
Q9BU76	otein 2 OS=Homo sapiens GN=MN	19.39	1	4	4	4	97.38	19.39	4	4					263	29.4	10.02
O60832	subunit 4 OS=Homo sapiens GN=D	11.67	7	5	5	6	97.29	11.67	5	6					514	57.6	9.42
B7Z2D8	ch sequence factor 1 OS=Homo sa	20.72	11	6	6	6	97.21	20.72	6	6					362	41.5	5.21
Q08ES8	tein 34 OS=Homo sapiens PE=2 S	22.03	4	4	4	5	94.95	22.03	4	5					177	20.1	9.60
A0A087WVV2	=Homo sapiens GN=RRBP1 PE=1	4.50	8	4	4	4	94.32	4.50	4	4					934	102.7	5.34
A0A087X1A5	homolog 1 OS=Homo sapiens GN	16.63	10	6	7	7	93.92	16.63	7	7					493	54.9	9.67
A6NHL2	S=Homo sapiens GN=TUBAL3 PE=	5.83	1	1	2	2	93.14	5.83	2	2					446	49.9	6.05
Q7L014	e DDX46 OS=Homo sapiens GN=D	4.95	4	5	5	5	91.73	4.95	5	5					1031	117.3	9.29
Q9BRL6	or 8 OS=Homo sapiens GN=SRSF	11.35	8	1	3	3	91.62	11.35	3	3					282	32.3	11.72
O14617	. OS=Homo sapiens GN=AP3D1 P	1.73	4	2	2	2	89.99	1.73	2	2					1153	130.1	8.48
Q7Z6E9	P6 OS=Homo sapiens GN=RBBP6	3.24	3	6	6	6	89.68	3.24	6	6					1792	201.4	9.64
E9PCY7	ein H OS=Homo sapiens GN=HNR	12.59	27	3	4	4	89.13	12.59	4	4					429	47.1	6.34
A8K3Y5	OS=Homo sapiens PE=2 SV=1 - [/	16.62	2	5	5	5	89.07	16.62	5	5					379	43.6	9.57
P83881	OS=Homo sapiens GN=RPL36A PE	26.42	7	2	5	5	88.11	26.42	5	5					106	12.4	10.58
Q53GL1	se 1 variant (Fragment) OS=Homo	19.00	3	4	4	5	87.55	19.00	4	5					279	31.9	8.87
Q96BA7	S=Homo sapiens PE=2 SV=1 - [C	6.79	6	4	4	4	87.45	6.79	4	4					722	79.7	7.87
B2R9I9	main 57 (U5 snRNP specific) (WDF	27.73	3	5	5	5	86.01	27.73	5	5					357	39.2	8.41
A0A024R7L5	ast), isoform CRA_b OS=Homo sap	8.05	8	7	7	7	85.73	8.05	7	7					1118	123.0	6.68
B2R4V2	nal protein L36a-like (RPL36AL), m	26.42	3	1	4	4	85.23	26.42	4	4					106	12.5	10.65
P46779	OS=Homo sapiens GN=RPL28 PE	20.44	5	4	4	4	84.73	20.44	4	4					137	15.7	12.02
P62805	no sapiens GN=HIST1H4A PE=1 S	21.36	2	2	2	2	83.27	21.36	2	2					103	11.4	11.36
Q7Z612	rotein P1 OS=Homo sapiens PE=2	57.52	4	3	3	6	81.78	57.52	3	6					113	11.4	4.36
P62861	0 OS=Homo sapiens GN=FAU PE=	18.64	2	2	2	3	81.20	18.64	2	3					59	6.6	12.15
P18077	OS=Homo sapiens GN=RPL35A PE	26.36	4	4	4	7	81.06	26.36	4	7					110	12.5	11.06
Q6PKG0	=Homo sapiens GN=LARP1 PE=1 S	6.30	8	6	6	6	80.42	6.30	6	6					1096	123.4	8.82
A0A087X0P6	o sapiens GN=IGKV2D-29 PE=4 S\	19.61	24	2	2	2	79.38	19.61	2	2					102	11.2	5.94
A8K800	omain containing 1 (BXDC1), mRN	21.90	4	7	7	7	78.96	21.90	7	7					306	35.6	9.99
Q53RD8	(Fragment) OS=Homo sapiens GN=	25.56	2	5	5	5	77.41	25.56	5	5					266	30.8	8.47
V9GZN0	55) (Fragment) OS=Homo sapiens	19.15	19	1	1	1	75.28	19.15	1	1					47	5.0	11.90
Q9BSQ6	S=Homo sapiens GN=RPL13A PE=	13.43	11	3	3	3	75.09	13.43	3	3					201	23.4	10.93
J3QLE5	tein N (Fragment) OS=Homo sapie	21.89	13	4	4	5	74.46	21.89	4	5					169	17.5	9.99
Q71DI3	mo sapiens GN=HIST2H3A PE=1 S	30.15	15	2	2	3	72.97	30.15	2	2	27.32	23.53	1	1	136	15.4	11.27
P62266	OS=Homo sapiens GN=RPS23 PE	23.78	5	3	3	3	72.60	23.78	3	3					143	15.8	10.49
Q5D862	mo sapiens GN=FLG2 PE=1 SV=1	1.46	1	3	3	3	71.31	0.92	2	2	20.09	0.54	1	1	2391	247.9	8.31
F8W7C6	S=Homo sapiens GN=RPL10 PE=	20.25	13	3	3	3	70.66	20.25	3	3					163	18.6	9.95
B4DL14	amma OS=Homo sapiens PE=2 S\	24.40	4	6	6	6	69.71	24.40	6	6					250	27.5	7.42
A8K7F6	on initiation factor 4A, isoform 1 (	15.52	28	5	5	5	69.11	15.52	5	5					406	46.1	5.48

Q14684	omolog B OS=Homo sapiens GN=	8.58	2	5	5	5	68.02	8.58	5	5					758	84.4	9.76
Q8WXA9	h protein 1 OS=Homo sapiens GN=	6.50	2	1	3	3	67.50	6.50	3	3					508	59.3	10.39
P68371	=Homo sapiens GN=TUBB4B PE=1	33.48	38	1	11	15	65.91	33.48	11	15					445	49.8	4.89
H0YAS6	gment) OS=Homo sapiens GN=PAI	27.81	5	1	3	6	65.70	27.81	3	5	46.34	14.20	1	1	169	17.9	9.19
D6RAN4	ent) OS=Homo sapiens GN=RPL9	13.74	8	2	2	2	65.60	13.74	2	2					182	20.9	10.20
B3KRJ9	to Splicing factor, arginine/serine-r	16.15	4	4	6	6	65.56	16.15	6	6					514	58.2	9.92
Q8N4P8	OS=Homo sapiens GN=GTPBP4 PE	9.18	6	5	5	5	65.52	9.18	5	5					632	73.7	9.55
P62316	n D2 OS=Homo sapiens GN=SNRI	24.58	3	3	3	3	65.15	24.58	3	3					118	13.5	9.91
Q13610	molog OS=Homo sapiens GN=PWI	21.16	9	8	8	10	64.88	21.16	8	10					501	55.8	4.77
X5CF57	sapiens GN=BRD2 PE=4 SV=1 - [	5.84	4	4	4	4	62.37	5.84	4	4					754	83.1	9.07
B4E0S6	-dependent RNA helicase DHX15 (	4.97	4	4	4	4	62.14	4.97	4	4					784	89.5	7.46
A8KAQ5	Da polypeptide (RNP antigen) (SNR	7.09	4	3	3	3	59.69	7.09	3	3					437	51.5	10.01
D3YTB1	ent) OS=Homo sapiens GN=RPL3	31.58	3	4	4	4	59.56	31.58	4	4					133	15.6	11.44
J3QLW7	NIP7 homolog OS=Homo sapiens (	40.00	5	3	3	3	59.25	40.00	3	3					110	12.4	8.27
P62318	n D3 OS=Homo sapiens GN=SNRI	15.08	1	2	2	2	58.79	15.08	2	2					126	13.9	10.32
D6R9K7	=Homo sapiens GN=RBM4 PE=1 S	27.33	9	4	4	4	58.63	27.33	4	4					150	17.0	5.97
P38919	III OS=Homo sapiens GN=EIF4A3	3.16	1	1	1	1	57.41	3.16	1	1					411	46.8	6.73
Q2NLD4	OS=Homo sapiens GN=PURA PE=2	3.51	4	1	1	1	56.96	3.51	1	1					285	32.0	7.05
A0A0A0MRQ5	o sapiens GN=PRDX1 PE=4 SV=1	11.34	6	1	1	1	56.38	11.34	1	1					97	10.7	8.72
H3BV80	omain 1 OS=Homo sapiens GN=R	5.69	6	1	1	1	55.37	5.69	1	1					211	24.5	11.90
P62854	OS=Homo sapiens GN=RPS26 PE	44.35	5	4	4	4	54.96	44.35	4	4					115	13.0	11.00
A8K201	GI-115 protein (CGI-115), mRNA (	5.02	2	1	1	1	54.88	5.02	1	1					259	28.9	5.38
G3V1B3	OS=Homo sapiens GN=RPL21 PE=	12.64	4	1	1	1	54.69	12.64	1	1					87	9.9	10.29
B0QZK4	3 (Fragment) OS=Homo sapiens G	15.02	4	2	2	2	54.28	15.02	2	2					253	28.5	9.88
A9UFC0	o sapiens GN=CASP14 PE=2 SV=1	9.09	2	2	2	2	54.04	9.09	2	2					242	27.6	5.34
A0A087WYV3	Homo sapiens GN=ZCCHC17 PE=1	17.32	7	3	3	3	53.83	17.32	3	3					179	20.4	9.89
Q9BTQ7	3 (Fragment) OS=Homo sapiens P	32.09	6	3	3	3	53.52	32.09	3	3					134	14.1	10.26
Q13151	tein A0 OS=Homo sapiens GN=HN	10.16	1	2	2	2	53.48	10.16	2	2					305	30.8	9.29
F5H018	agment) OS=Homo sapiens GN=F	14.57	6	3	3	3	53.37	14.57	3	3					199	22.5	8.73
D3DSF7	CRA_b OS=Homo sapiens GN=SO	2.18	2	1	1	1	52.93	2.18	1	1					689	72.5	4.49
P25705	ondrial OS=Homo sapiens GN=AT	13.20	8	6	6	6	52.45	13.20	6	6					553	59.7	9.13
Q5JRI1	r 10 OS=Homo sapiens GN=SRSF1	29.07	4	3	4	5	52.23	29.07	4	5					172	20.9	10.48
B4DQI6	pha OS=Homo sapiens GN=TRA2	8.89	2	2	2	2	51.88	8.89	2	2					180	20.6	10.17
H0YB22	nent) OS=Homo sapiens GN=RPS1	35.00	4	3	3	3	51.36	35.00	3	3					120	12.9	9.85
Q8TEZ9	ent) OS=Homo sapiens PE=2 SV=	2.40	3	1	1	1	50.42	2.40	1	1					500	57.0	5.10
B4DKT5	n factor 2 mRNA-binding protein 2	8.33	3	1	2	2	50.19	8.33	2	2					300	33.3	8.75
P56537	factor 6 OS=Homo sapiens GN=E	17.14	2	3	3	3	49.56	17.14	3	3					245	26.6	4.68
P78362	S=Homo sapiens GN=SRPK2 PE=1	5.52	9	3	4	4	49.42	5.52	4	4					688	77.5	4.97
P81605	omo sapiens GN=DCD PE=1 SV=2	22.73	1	3	3	6	49.37	22.73	3	3	83.36	20.00	2	3	110	11.3	6.54
A8K9J7	S=Homo sapiens PE=2 SV=1 - [A	8.73	20	1	1	1	48.40	8.73	1	1					126	14.0	10.32
O60382	Homo sapiens GN=KIAA0324 PE=	0.50	2	1	1	1	48.12	0.50	1	1					1791	191.2	11.93
Q9UKD2	log OS=Homo sapiens GN=MRTO	17.15	2	4	4	4	47.61	17.15	4	4					239	27.5	8.29
B4E3J6	OS=Homo sapiens PE=2 SV=1 - [	7.17	2	2	2	2	47.27	7.17	2	2					223	26.6	9.54
J3KSS0	OS=Homo sapiens GN=RPL26 PE=	14.29	9	1	1	1	47.23	14.29	1	1					63	7.6	11.00
P49711	CF OS=Homo sapiens GN=CTCF P	4.13	1	3	3	3	46.61	4.13	3	3					727	82.7	6.96
F5H1V1	ent) OS=Homo sapiens GN=ARF3	27.50	16	1	1	1	46.39	27.50	1	1					40	4.3	10.13
P55769	Homo sapiens GN=NHP2L1 PE=1	15.63	2	2	2	2	45.88	15.63	2	2					128	14.2	8.46
B4DVF8	tein L OS=Homo sapiens GN=HNI	10.61	8	2	2	2	45.82	10.61	2	2					179	19.8	7.12
A2I829	ment) OS=Homo sapiens GN=NKI	1.50	4	1	1	1	45.72	1.50	1	1					599	67.4	8.87
C9JN15	ragment) OS=Homo sapiens GN=	10.98	10	3	3	3	45.49	10.98	3	3					246	27.4	9.32
D6RB09	ent) OS=Homo sapiens GN=RPS3	24.74	14	5	5	5	45.38	24.74	5	5					194	22.6	9.76

P25398	OS=Homo sapiens GN=RPS12 PE	25.76	1	3	3	4	44.17	25.76	3	4					132	14.5	7.21
O00567	=Homo sapiens GN=NOP56 PE=1	19.70	6	9	9	9	43.43	19.70	9	9					594	66.0	9.19
B4DZC3	onuclease 2 (EC 3.1.11.-) OS=Ho	4.46	3	3	3	3	43.05	4.46	3	3					896	102.4	7.64
A5D904	S=Homo sapiens GN=RPS9 PE=2	17.70	5	2	2	3	42.80	17.70	2	3					113	13.2	10.45
A0A024R836	S=Homo sapiens GN=MGC11257	28.66	5	3	3	3	42.32	28.66	3	3					157	18.5	9.23
A8K4Z6	OS=Homo sapiens PE=2 SV=1 - [	4.68	2	3	3	3	41.79	4.68	3	3					619	70.5	9.83
A0A024QZH6	., isoform CRA_a OS=Homo sapier	0.76	2	1	1	1	41.68	0.76	1	1					1312	139.2	9.25
H7C4V2	otein (Fragment) OS=Homo sapier	3.10	4	1	1	1	41.53	3.10	1	1					290	33.7	5.14
O00373	omo sapiens PE=4 SV=1 - [O0037	6.53	17	2	2	2	40.82	6.53	2	2					337	39.8	9.39
A0A024R1X8	_a OS=Homo sapiens GN=JUP PE-	8.32	6	4	4	4	40.48	8.32	4	4					745	81.7	6.14
H3BTN5	OS=Homo sapiens GN=PKM PE=	11.34	14	5	5	5	40.38	8.04	4	4	34.70	3.30	1	1	485	53.0	6.84
B4DRA2	le protein (Fragment) OS=Homo s	1.08	4	1	1	1	40.32	1.08	1	1					923	93.7	8.94
P10599	omo sapiens GN=TXN PE=1 SV=3	8.57	1	1	1	2	39.91	8.57	1	1	51.75	8.57	1	1	105	11.7	4.92
M0R0P1	(Fragment) OS=Homo sapiens GN	24.12	9	3	3	3	39.87	24.12	3	3					228	24.5	10.11
P62304	n E OS=Homo sapiens GN=SNRPE	39.13	2	2	2	3	39.66	39.13	2	2	43.48	27.17	1	1	92	10.8	9.44
Q9H4N9	equence OS=Homo sapiens PE=2	1.99	6	1	1	1	39.46	1.99	1	1					351	39.2	7.69
Q13823	n 2 OS=Homo sapiens GN=GNL2 F	2.46	3	2	2	2	39.34	2.46	2	2					731	83.6	9.25
P48444	S=Homo sapiens GN=ARCN1 PE=	2.15	3	1	1	1	39.20	2.15	1	1					511	57.2	6.21
P62280	OS=Homo sapiens GN=RPS11 PE	17.72	3	2	2	2	39.06	17.72	2	2					158	18.4	10.30
P17096	HMG-Y OS=Homo sapiens GN=HM	14.95	3	1	1	2	38.85	14.95	1	2					107	11.7	10.32
A2KBC1	ment) OS=Homo sapiens PE=2 SV	3.81	1	1	1	1	38.46	3.81	1	1					236	25.1	8.12
A0A024R0A2	omo sapiens GN=RPL37 PE=3 SV	8.54	3	1	1	1	38.32	8.54	1	1					82	9.5	11.28
Q02413	omo sapiens GN=DSG1 PE=1 SV=	8.67	1	7	7	9	38.29	5.34	5	5	37.73	5.62	4	4	1049	113.7	5.03
B2RDV4	OS=Homo sapiens PE=2 SV=1 - [	2.47	2	1	1	1	38.05	2.47	1	1					364	40.4	8.03
P35268	OS=Homo sapiens GN=RPL22 PE	30.47	8	2	2	2	37.99	30.47	2	2					128	14.8	9.19
M0QY97	4 (Fragment) OS=Homo sapiens	5.27	2	3	3	3	37.94	5.27	3	3					910	95.5	7.33
D6RC52	2 (Fragment) OS=Homo sapiens	21.97	5	2	2	2	37.91	21.97	2	2					132	15.0	9.25
Q96HX7	S=Homo sapiens GN=HSP90AA1	6.40	4	1	1	3	37.63	6.40	1	1	40.17	6.40	1	2	422	49.2	5.58
Q9BZU1	Homo sapiens PE=2 SV=1 - [Q9B	15.19	4	1	1	1	37.54	15.19	1	1					79	9.0	11.53
A0A087WWP8	S=Homo sapiens GN=RSBN1 PE=	2.79	2	2	2	2	37.31	2.79	2	2					754	84.9	8.50
Q5QPM0	ent) OS=Homo sapiens GN=RALY	5.23	5	1	1	1	37.24	5.23	1	1					172	18.7	10.02
O14889	ment) OS=Homo sapiens PE=2 S	12.00	45	1	1	1	37.19	12.00	1	1					75	8.6	9.48
J3KSY7	S=Homo sapiens GN=CASC3 PE=	4.93	4	1	1	1	37.12	4.93	1	1					223	25.7	9.17
E5RJX2	S=Homo sapiens GN=RPS20 PE=	11.48	2	1	1	1	37.00	11.48	1	1					61	6.8	10.35
Q9Y2X3	=Homo sapiens GN=NOP58 PE=1	13.23	4	5	5	5	36.98	13.23	5	5					529	59.5	8.92
P63241	tor 5A-1 OS=Homo sapiens GN=E	15.58	2	1	1	3	36.80	15.58	1	1	56.09	15.58	1	2	154	16.8	5.24
Q96B39	S=Homo sapiens GN=TRMT61A F	11.58	3	3	3	3	36.10	11.58	3	3					190	20.4	7.25
Q9NQ55	log OS=Homo sapiens GN=PPAN F	6.77	3	3	3	3	35.86	6.77	3	3					473	53.2	10.13
H0YEN5	ent) OS=Homo sapiens GN=RPS2	10.77	15	2	2	2	35.77	10.77	2	2					195	21.1	9.83
A8K651	inding protein (C1QBP), nuclear g	4.96	2	1	1	1	35.53	4.96	1	1					282	31.4	4.84
B3KM36	AG family molecular chaperone re	4.27	3	1	1	1	35.01	4.27	1	1					211	23.7	6.70
C9JXB8	S=Homo sapiens GN=RPL24 PE=	7.44	3	1	1	1	34.88	7.44	1	1					121	14.4	11.31
Q5JRC6	=Homo sapiens GN=PHF6 PE=1 S	20.37	2	5	5	5	34.85	20.37	5	5					324	36.4	9.03
P01625	ion Len OS=Homo sapiens PE=1 S	7.89	7	1	1	1	34.45	7.89	1	1					114	12.6	7.93
B4DW52	tin, cytoplasmic 1 OS=Homo sapie	21.04	73	4	4	4	34.37	3.17	1	1	20.83	17.87	3	3	347	38.6	5.35
Q96CA8	S=Homo sapiens GN=CDC2L2 PE	1.72	13	1	1	1	34.14	1.72	1	1					464	52.4	5.08
I3L239	(Fragment) OS=Homo sapiens G	4.65	10	1	1	1	33.81	4.65	1	1					215	24.9	9.31
A8K964	mosome associated protein (PNN),	3.91	4	3	3	3	33.54	3.91	3	3					717	81.5	7.37
Q09161	nit 1 OS=Homo sapiens GN=NCBP	1.77	1	1	1	1	33.22	1.77	1	1					790	91.8	6.43
Q9BQR2	nerase I (Fragment) OS=Homo sa	8.86	6	1	1	1	33.05	8.86	1	1					158	18.6	9.13
I3L3P7	S=Homo sapiens GN=RPS15A PE	22.00	4	2	2	2	32.59	22.00	2	2					100	11.5	10.15

A0A024R2Z6	r), isoform CRA_b OS=Homo sapie	5.21	2	3	3	3	32.16	5.21	3	3					537	60.5	8.79
I6L957	omo sapiens GN=HNRNPA2B1 PE=	7.23	5	2	2	2	32.06	7.23	2	2					249	28.4	4.86
A0A087WUI1	2 OS=Homo sapiens GN=RBMX2 P	9.09	3	2	2	2	30.74	9.09	2	2					275	31.6	9.45
H7C109	57 (Fragment) OS=Homo sapiens (	2.41	3	1	1	1	30.42	2.41	1	1					665	75.0	8.09
P01767	ion BUT OS=Homo sapiens PE=1 S	16.52	1	1	1	3	30.35	16.52	1	2	0.00	16.52	1	1	115	12.4	9.25
Q8WXF0	r 12 OS=Homo sapiens GN=SRSF1	14.56	1	2	3	3	30.10	14.56	3	3					261	30.5	11.69
F8W0G4	ent) OS=Homo sapiens GN=PCBP2	8.23	9	1	1	1	29.76	8.23	1	1					158	16.6	7.77
B5BTZ8	ptide B'' OS=Homo sapiens GN=SI	4.89	2	1	1	1	29.72	4.89	1	1					225	25.4	9.72
J3QLI9	n D1 OS=Homo sapiens GN=SNRP	44.00	3	2	2	2	29.71	44.00	2	2					75	8.4	11.84
Q9BRT6	S=Homo sapiens GN=LLPH PE=2	12.40	1	1	1	2	28.93	12.40	1	2					129	15.2	10.37
Q5T4L4	OS=Homo sapiens GN=RPS27 PE=	19.70	6	1	1	1	28.89	19.70	1	1					66	7.4	7.24
Q9NZT1	S=Homo sapiens GN=CALML5 PE=	15.75	1	1	1	1	28.62	15.75	1	1					146	15.9	4.44
C9JP48	Fragment) OS=Homo sapiens GN=	19.57	15	2	2	2	27.68	19.57	2	2					138	15.8	5.15
Q70T18	gment) OS=Homo sapiens PE=2 S	5.73	11	1	1	1	27.59	5.73	1	1					157	16.1	9.00
C9JB90	nent) OS=Homo sapiens GN=RAB(	22.45	77	1	1	1	27.09	22.45	1	1					49	5.9	4.82
P62633	otein OS=Homo sapiens GN=CNBF	15.82	2	2	2	3	26.63	15.82	2	3					177	19.4	7.71
A8K3M9	tor, arginine/serine-rich 9 (SFRS9)	9.50	4	2	2	2	26.36	9.50	2	2					221	25.5	8.65
E5RJN7	molog 2 (Fragment) OS=Homo sap	6.56	16	1	2	2	25.84	6.56	2	2					320	34.8	9.52
P17026	=Homo sapiens GN=ZNF22 PE=1	9.82	6	2	2	2	25.73	9.82	2	2					224	25.9	10.04
Q59H78	(Fragment) OS=Homo sapiens PE	4.80	10	1	1	1	24.37	4.80	1	1					583	61.4	7.42
Q9Y6Q3	og OS=Homo sapiens GN=ZFP37 P	1.59	1	1	1	1	24.08	1.59	1	1					630	71.2	9.09
Q8TCJ2	ansferase subunit STT3B OS=Hom	1.45	1	1	1	1	23.61	1.45	1	1					826	93.6	8.91
Q9BQQ5	S=Homo sapiens GN=L27a PE=3 S	16.98	5	2	2	2	23.25	16.98	2	2					106	12.0	11.46
P62306	n F OS=Homo sapiens GN=SNRPF	24.42	1	2	2	2	20.97	24.42	2	2					86	9.7	4.67
Q9NUD5	rotein 3 OS=Homo sapiens GN=Z(	7.67	1	3	3	3	20.32	7.67	3	3					404	43.6	8.53
I3L1L3	t) OS=Homo sapiens GN=MYBBP1	3.43	3	4	4	4	0.00	3.43	4	4					1252	140.2	9.26
B7Z1V7	0 protein, mitochondrial OS=Hom	13.73	9	4	4	4	0.00	13.73	4	4					437	47.3	6.61
B8ZZK4	OS=Homo sapiens GN=RPL31 PE=	30.38	7	3	3	3	0.00	30.38	3	3					79	9.0	10.89
A8K3W9	OS=Homo sapiens PE=2 SV=1 - [A	10.22	5	2	2	3	0.00	10.22	2	2	59.39	7.46	1	1	362	41.6	5.07
P49411	drial OS=Homo sapiens GN=TUFM	5.31	1	2	2	2	0.00	5.31	2	2					452	49.5	7.61
Q6ZN17	S=Homo sapiens GN=LIN28B PE=	13.20	2	2	2	2	0.00	13.20	2	2					250	27.1	8.91
Q5T6W5	tein K OS=Homo sapiens GN=HNR	4.91	9	2	2	2	0.00	4.91	2	2					428	47.5	5.63
B4DZQ7	ent RNA helicase DDX17 (EC 3.6.1	5.00	11	2	2	2	0.00	5.00	2	2					420	47.5	7.66
Q49AN9	mo sapiens GN=SNRPG PE=2 SV=	18.75	4	1	1	1	0.00	18.75	1	1					64	7.1	7.18
O95400	protein 2 OS=Homo sapiens GN=C	4.11	1	1	1	1	0.00	4.11	1	1					341	37.6	4.61
Q96BK5	nhibitor 1 OS=Homo sapiens GN=I	2.74	1	1	1	1	0.00	2.74	1	1					328	37.0	9.60
Q9UJA5	atalytic subunit TRM6 OS=Homo s	3.22	1	1	1	1	0.00	3.22	1	1					497	55.8	7.55
D3DP16	CRA_a OS=Homo sapiens GN=FG(	5.99	5	1	1	1	0.00	5.99	1	1					334	37.7	6.29
A0A087WVZ1	gment) OS=Homo sapiens GN=DD	10.14	5	1	1	1	0.00	10.14	1	1					138	14.8	9.98
A0A087WWP4	S=Homo sapiens GN=RBM15 PE=	1.50	2	1	1	1	0.00	1.50	1	1					933	102.1	9.94
B4DH82	tein homolog (Fragment) OS=Hom	0.77	1	1	1	1	0.00	0.77	1	1					1299	143.1	8.32
D6RDJ3	RPAC1 (Fragment) OS=Homo sapi	14.52	1	1	1	1	0.00	14.52	1	1					124	14.2	5.27
Q6N014	(Fragment) OS=Homo sapiens GN=	1.86	4	1	1	1	0.00	1.86	1	1					590	67.3	10.59
B3KN79	Da 2'-5'-oligoadenylate synthetase	1.95	2	1	1	1	0.00	1.95	1	1					514	59.2	7.87
Q05BI1	OS=Homo sapiens GN=CD3EAP PE	3.57	4	1	1	1	0.00	3.57	1	1					392	42.0	9.11
B4DRJ7	in containing 5 (PAPD5), transcrip	3.32	9	1	1	1	0.00	3.32	1	1					271	29.6	9.10
V9GYG0	=Homo sapiens GN=SLC25A4 PE=1	5.29	6	1	1	1	0.00	5.29	1	1					208	22.9	9.54
L8EC67	OS=Homo sapiens GN=DGKK PE=4	17.86	1	1	1	1	0.00	17.86	1	1					56	6.5	8.88
I6L8B7	rmal OS=Homo sapiens GN=FABP5	22.77	2	2	2	2					34.21	22.77	2	2	101	11.2	6.07
P04406	rogenase OS=Homo sapiens GN=(	19.70	4	2	2	2					27.36	19.70	2	2	335	36.0	8.46
F8VV32	omo sapiens GN=LYZ PE=1 SV=1 -	8.65	2	1	1	1					46.58	8.65	1	1	104	11.5	9.07

P22528	o sapiens GN=SPRR1B PE=1 SV=2	17.98	3	1	1	1					36.13	17.98	1	1	89	9.9	8.48
Q8TDB1	F33V (Fragment) OS=Homo sapien	15.79	8	1	1	1					34.61	15.79	1	1	38	4.0	7.18
P05109	omo sapiens GN=S100A8 PE=1 SV	11.83	1	1	1	1					32.53	11.83	1	1	93	10.8	7.03
B2R577	S=Homo sapiens PE=2 SV=1 - [B2	27.78	2	1	1	1					25.73	27.78	1	1	90	10.1	5.91
P31151	omo sapiens GN=S100A7 PE=1 SV	10.89	1	1	1	1					0.00	10.89	1	1	101	11.5	6.77
P22531	OS=Homo sapiens GN=SPRR2E PE	25.00	7	1	1	1					0.00	25.00	1	1	72	7.8	8.31
Q6IBT3	omo sapiens GN=CCT7 PE=2 SV=1	4.05	1	1	1	1					0.00	4.05	1	1	543	59.3	7.65
